# Ultrasound-promoted two-step synthesis of 3-arylselenylindoles and 3-arylthioindoles as novel combretastatin A-4 analogues

**DOI:** 10.1038/srep23986

**Published:** 2016-04-05

**Authors:** Zhiyong Wen, Xiaona Li, Daiying Zuo, Binyue Lang, Yang Wu, Mingyang Jiang, Huizhuo Ma, Kai Bao, Yingliang Wu, Weige Zhang

**Affiliations:** 1 Key Laboratory of Structure-Based Drug Design and Discovery, Ministry of Education, Shenyang Pharmaceutical University, 103 Wenhua Road, Shenhe District, Shenyang 110016 (China); 2 Department of Pharmacology, Shenyang Pharmaceutical University, 103 Wenhua Road, Shenhe District, Shenyang 110016 (China); 3 Division of Hematology/ Oncology, Department of Medicine, Beth Israel Deaconess Medical Center and Harvard Medical School, Boston, MA 02215 (USA)

## Abstract

A series of 3-(3′-hydroxy-4′-methoxyphenyl)selenyl-5,6,7-trimethoxy-1*H*-indoles and 3-(3′-hydroxy-4′-methoxyphenyl)thio-5,6,7-trimethoxy-1*H*-indoles were obtained as a new class of combretastatin A-4 (CA-4) analogues *via* a convenient ultrasound (US)-assisted two-step process involving 3-selenenylation/sulfenylation followed by *O*-deallylation. With the assistance of US irradiation, both the reaction rates and yields of selenenylation, sulfenylation and *O*-deallylation could be significantly improved. A comparison of the reaction rates of *O*-deallylation and ester reduction demonstrated that *O*-deallylation was more sensitive to US irradiation. Finally, these products were evaluated for their antiproliferative activities, and most of them showed moderate to potent activities against three human cancer cell lines *in vitro*.

CombretastatinA-4 (CA-4, **1a**, Fig. 1), a natural stilbene isolated from the South African tree *Combretum caffrum*, is a powerful inhibitor of tubulin polymerization that displays cytotoxic and antivascular activities by binding at the colchicine site of tubulin^1^. CA-4P (**1b**), the water-soluble pro-drug of CA-4, has shown promising results in human cancer clinical trials^2^. The structure-activity relationship (SAR) study of CA-4 indicated that the presence of a *cis*-double bond and 3,4,5-trimethoxyphenyl ring-A is essential for its anti-tubulin activity, and the introduction of a hydroxyl group to the C′-3 position in ring-B was found to strongly enhance its activity. Replacement of ring-A and ring-B with other aromatic or heteroaromatic systems, and swapping the positions of ring-A and ring-B are very effective strategies for designing CA-4 analogues^3^,^4^,^5^,^6^,^7^,^8^,^9^.

In the last few years, a number of CA-4 analogues with potent activities have been developed. Among these analogues, indole group-containing compounds (**2**, **3, 4**) have been reported to show potent antitubulin and antiproliferative activities^3^,^4^,^10^,^11^. Recently, we reported the development of a set of 3-arylselenylindoles (**5**) as CA-4 analogues, which also exhibited potent activities in the tubulin polymerization inhibition and against three human cancer cell lines^12^. These results led us to start a pharmacophore exploration and optimization effort around the 3-arylselenyl-indoles and the bioisosteric 3-arylthio-indoles, moving the trimethoxyl groups to the indoline moiety, to afford the isomeric 5,6,7-trimethoxyindole derivatives with a general structure (**6**) as shown in Fig. 1.

Most protocols for the synthesis of 3-arylthioindoles and 3-arylselenylindoles are limited by the instability and the complex synthetic process of activated sulfur or selenium reagents^13^,^14^,^15^,^16^,^17^. A previously reported method for direct synthesis of 3-arylthioindoles and 3-arylselenylindoles using iron (III) and iodine as the catalysts suffered from long reaction time^18^. Previously, we improved this method by using microwave irradiation to shorten the reaction times and increase the yields^12^. Ultrasound (US) is becoming a widely used laboratory and industrial technique that offers a green energy source for organic synthesis and has been proven to be a very useful tool in enhancing the reaction rates in a variety of reacting systems under quite mild conditions (room temperature)^19^,^20^,^21^,^22^. It was found that the sonochemical effect of US waves mainly comes from acoustic cavitation, which provides enough kinetic energy to drive reactions to completion in shorter durations^19^,^20^. However, to the best of our knowledge, only a very limited number of studies have reported the synthesis of 3-arylselenyl- and 3-arylthio-indoles under US conditions^23^.

In our effort to search for antitubulin/antiproliferative agents, a new series of 3-arylselenyl- and 3-arylthio-indoles were designed as CA-4 analogues, in which 5,6,7-trimethoxy indole was employed as ring-A, 3′-hydroxyl-4′-methoxyphenyl was employed as ring-B and selenium or sulfur atoms were used as the substitutions for the *cis*-double bond of CA-4. In addition, several substituents were introduced to the N-1 and C-2 positions of the indole rings. All target compounds were obtained *via* an US-assisted two-step process involving 3-selenenylation/sulfenylation followed by a rapid selective *O*-deallylation. To verify the US efficiency in this two-step protocol, a comparative study between reactions performed under non-ultrasound (non-US) and US conditions was carried out. Finally, we evaluated the antiproliferative activities of the newly developed compounds against three human cancer cell lines. And the effects of the most potent compound, **10a,** on the inhibition of tubulin assembly were tested *in vitro* by immunofluorescence studies.

## Results and Discussion

### Chemistry

Based on our previous study on the synthesis of 3-arylselenylindoles under microwave irradiation conditions^12^ (at 80 °C), 3-selenenylation/sulfenylation of indoles was tested by using US irradiation under quite mild conditions (at room temperature). The synthesis of **9a** from 5,6,7-trimethoxy-2-methyl-1*H*-indole^24^,^25^,^26^,^27^ (**7a**) and 1,2-bis(3-(allyloxy)-4-methoxyphenyl) diselenide^12^ (**8a**) in the presence of 20 mol% of FeCl_3_ and 1 mol% of iodine was studied initially, and the results are summarized in Table 1. As shown in entry 1, **9a** was isolated in only 67% yield when the reaction was refluxed in acetonitrile for 24 h by thermal heating (oil bath). In contrast, **9a** was obtained in moderate yield (52%) by US irradiation at room temperature for 3 h (entry 2). A clear improvement in yield was observed when the reaction time was prolonged to 6 h (entry 3). However, further extending the reaction time under US conditions did not show apparent benefits (entry 4). In addition, only THF showed a comparable efficiency for this reaction after screening of the other solvents (entries 5–8). The optimized conditions were developed when acetonitrile was chosen as the solvent with the assistance of US irradiation for 6–9 h at room temperature.

Next, the optimized reaction conditions were used for the synthesis of compounds **9b**–**h** (Table 2). By changing the methyl group at the C-2 position of compound **9a** to hydrogen, many other indoles with various substrates at the N-1 position successfully underwent 3-selenenylation to furnish the desired products. The results in entries 1–4 clearly indicate that US conditions generated higher yields and much faster reaction rates than non-US conditions for the 3-selenenylation reaction. These reaction conditions were further utilized for the synthesis of thioindoles **9f–h** using various indoles with disulphide^28^,^29^ (**8b**), which allowed the reactions to proceed smoothly and generate the desired products in moderate yields (entries 5–7).

Allyl groups are widely used in organic synthesis as important protecting groups of alcohols due to the availability and stability of the corresponding allyl derivatives. Many methodologies have been documented in terms of deprotection of allyl ethers^30^,^31^,^32^,^33^. In our case, an allyl group was used as the protecting group of the 3′-phenolic hydroxyl group. The *O*-deallylation reactions to furnish the 3′-phenolic hydroxyl substituted target compounds were initiated by using TiCl_4_, an efficient *O*-deallylation agent^34^, at −20 °C in dry dichloromethane. Unfortunately, **9a** decomposed under these conditions and no deallylation product (**10a**) was observed.

Pd(PPh_3_)_4_/NaBH_4_ was reported to be a useful system for the deprotection of *O*-allyl under neutral conditions^35^,^36^. When we performed the *O*-deallylation reaction by following this procedure using 1 mol% of Pd(PPh_3_)_4_ and an excess of sodium borohydride (8 equivalents) at room temperature for 30 h, **10a** could be isolated in 73% yield (Table 3, entry 2). To our delight, the reaction rates and yields were significantly improved by US irradiation. When this reaction was performed under US irradiation for 3 h, **10a** was obtained in 64% yield (entry 4). Furthermore, the yield was increased to 90% after 3 h US irradiation with 10 mol% of Pd(PPh_3_)_4_ (entry 7). In contrast, a lower reaction yield and rate were obtained when the reaction was performed using the same amount of Pd(PPh_3_)_4_ under non-US conditions (entry 8).

Then, the optimized conditions were applied for the synthesis of compounds **10b–h** (Table 4). Under US conditions, all the *O*-deallylations were performed successfully within a shorter reaction time and provided higher yields compared with reactions conducted under non-US conditions. The reducible functional groups, including amide, N-benzyl and halogen (Cl), showed good tolerance to these reaction conditions. However, when the deallylation of **9d** and **9g** were performed under US conditions for more than 5 h, compounds **11** and **12** were obtained as the predominant products respectively, in which the allyl ethers were cleaved and the ester groups were simultaneously reduced. When these reactions were carried out under non-US conditions at r.t. until the starting materials **9d** and **9g** were fully consumed (over 10 h), only compounds **11** and **12** were obtained, respectively. Thus, we believed that there was a significant rate difference between the *O*-deallylation and ester reduction reactions under US conditions.

**Figure 2:**
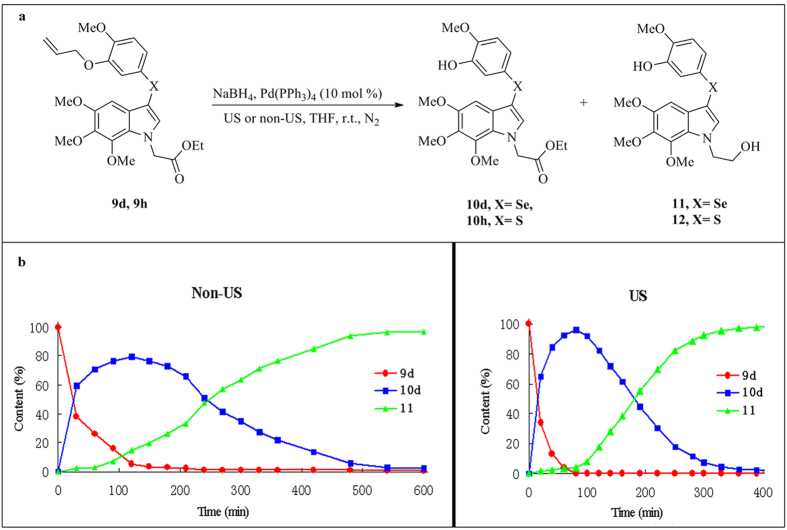
Comparison of *O*-Deallylation and ester reduction of **9d**, **9g** under non-US and US conditions. (**a**) Experiments of *O*-deallylation and ester reduction of **9d**, **9g**. (**b**) HPLC analysis of *O*-deallylation and ester reduction of **9d** under non-US and US conditions.

To investigate the rate differences between the *O*-deallylation and the ester reduction, the reactions of **9d** under non-US and US conditions were detected by high performance liquid chromatography (HPLC). As shown in Fig. 2(b), when the reaction was performed under non-US conditions for 120 min, 80% of **10d** in the reaction mixture was observed along with 15% of **11** as the reduction product. Moreover, the lower reaction rate under non-US conditions required a reaction time of at least 10 h for the full consumption of **9d**, and all the ester groups in **10d** had been reduced to hydroxyl groups. However, under US conditions, substrate **9d** was quantitatively converted into **10d** within 80 min (95% yield). US irradiation for more than 7 h resulted in the full reduction of the ester group of **10d**. Compared with the yields of **10d** and **11** at 80 min under non-US conditions, US irradiation significantly increased the rate of *O*-deallylation. It is worth noting that this magnified rate difference between the US promoted *O*-deallylation and ester reduction made it possible to selectively synthesize the desired compounds as the predominant products.

**Figure 3:**
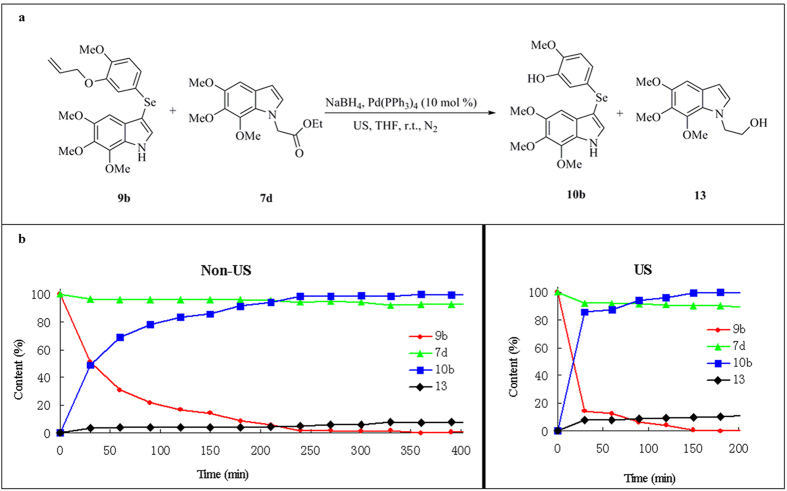
Comparison of *O*-deallylation of **9b** and ester reduction of **7d** under non-US and US conditions. (**a**) Experiments of **9b** and **7d**. (**b**) HPLC analysis of *O*-deallylation of **9b** and ester reduction of **7d** under non-US and US conditions.

To further illuminate the rate differences between the *O*-deallylation and reduction of ester under US conditions, an intermolecular competition experiment using an equimolar mixture of **9b** and **7d** was carried out under US and non-US conditions, respectively (Fig. 3). The results demonstrated that under US conditions, the *O*-deallylation rate of **9b** was approximately 1.7 fold faster than that performed under non-US conditions within 30 min of the reaction, while the reduction of **7d** showed no significant difference between the US and non-US conditions. These results were in agreement with those obtained from the intramolecular competition experiment under US conditions.

### Antiproliferative activity assay

To evaluate the antiproliferative activity, the target compounds and reference compound CA-4 were screened against three human cancer cell lines (gastric adenocarcinoma SGC-7901 cells, mouth epidermal carcinoma KB cells and fibrosarcoma HT-1080 cells) using a standard MTT assay (Table 5). The reported IC_50_ values are the average of at least three independent experiments. Generally, the target compounds in this study were less potent than the ones with the trimethoxyl groups on the 3-arylselenyl ring in our previous study^17^, except **10a**. The 3-arylselenylindoles were more active than the bioisosteric 3-arylthioindole analogues, but the exact reason for these observations needs to be further investigated. The most active compound, **10a**, was found to exhibit activity comparable to that of CA-4 and showed IC_50_ values in the nanomolar range against all three of the cell lines. The introduction of various substituents to the N-1 position of the indole ring also tended to reduce potency when comparing the activities of **10b** with **10c**, **10d** and **10e**. In addition, removal of the methyl group at the C-2 position of **10a** resulted in a loss of activity (**10b**). It reminds us that modification at the C-2 position may have a direct effect on the activity. Future study of the 3-arylselenylindoles will focus on the C-2 substituents variations including -Et, -OMe, -CHO and others.

### Immunofluorescence studies

**Figure 4:**
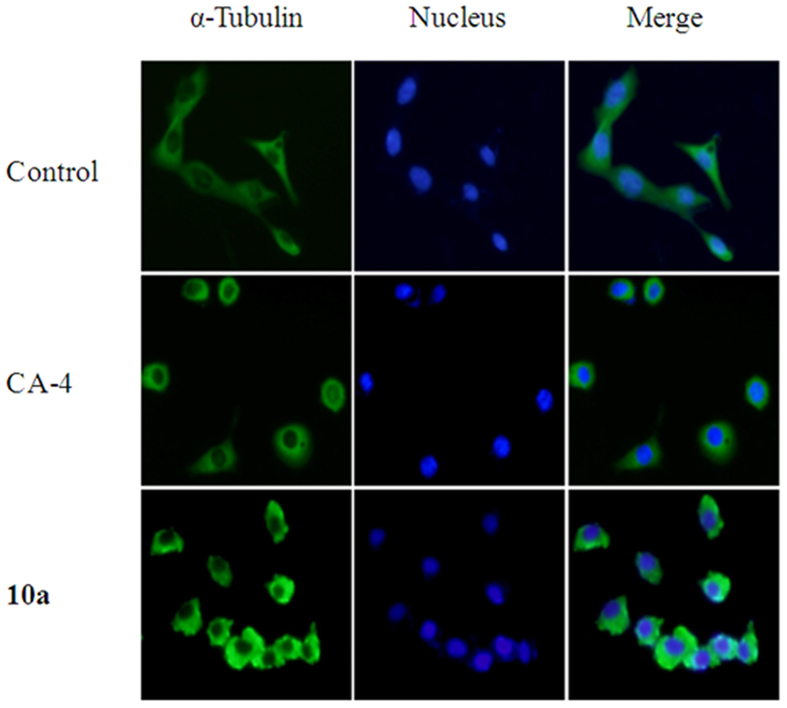
Compound **10a** and CA-4 induce microtubules depolymerization in HT-1080 cells. HT-1080 cells were treated with CA-4 (11 nM) or compound **10a** (14 nM) for 24 h. Cells were fixed and stained with monoclonal α-tubulin (green) and counterstained with DAPI (blue). Immunofluorescence was detected using a fluorescence microscope (Olympus, Tokyo, Japan, bar scale 50 μm).

We further investigated the capacity of the most potent compound, **10a**, to alter the microtubule network in HT-1080 cell by tubulin immunostaining and compared the observations to those of the reference compound CA-4 (Fig. 4). Confocal analysis showed that the microtubule network exhibited normal arrangement and organization (slim and fibrous) in the control cells. Exposure to 11 nM of CA-4 for 24 h led to a complete loss of microtubule formation (microtubules became short and wrapped around the nucleus) and strongly affected the cell shape (turned round); treatment with 14 nM of compound **10a** produced results similar to those of CA-4-induced microtubule and cell shape changes. These results confirmed that **10a** exerted similar effects to CA-4 on the microtubule network, suggesting that **10a** is most likely targeting tubulin.

In summary, a convenient and efficient US-assisted two-step process was developed for the synthesis of novel combretastatin A-4 analogues, 3-(3′-hydroxy-4′-methoxyphenyl)selenyl-5,6,7-trimethoxy-1*H*-indoles and 3-(3′- hydroxy-4′-methoxyphenyl)thio-5,6,7-trimethoxy-1*H*-indoles, which involved US-promoted 3-selenenylation/sulfenylation followed by a *O*-deallylation. Compared with conventional methods used to prepare these compounds, US irradiation induced a remarkable acceleration in the reaction rates and generated the products in higher yields. Moreover, US irradiation significantly increased the *O*-deallylation rate, while having almost no evident influence on the reduction of the esters. This magnified rate difference made it possible to selectively synthesize *O*-deallylic or *O*-deallylic and ester reduced compounds as predominant products. The synthesized target compounds were also evaluated for their antiproliferative activities against three human cancer cell lines *in vitro*, and most of them exhibited moderate to potent activities. An immunofluorescence study of compound **10a** revealed that its target was most likely tubulin. Further studies of seleniferous indoles substituted at the C-2 position are currently in process.

## Methods

### Reagents and equipment

All the solvents and chemical materials were commercially available, and were used without further purification. Silica gel GF254 and silica gel H (200–300 mesh) from Qingdao Haiyang Chemical Company were used for preparative thin-layer chromatography and column chromatography, respectively. US irradiation was performed in an ultrasonic cleaner (KQ-400KDE, made in Kunshan Ultrasonic Equipment Co., Ltd.) with frequency of 25 kHz and a nominal power of 400 W at 25–30 ^o^C. The content of diselenide (**8a**) was analyzed by HPLC (HPLC-LC-20AT, Shimadzu) using SPD-20A UV as the detector and 80% methanol in water as mobile elution. ^1^H and ^13^C NMR spectra were recorded in CDCl_3_ or DMSO-*d*_6_ (TMS as internal standard) using a Bruker Avance 300, 400, or 600 spectrometer (^1^H at 300, 400 or 600 MHz, ^13^C at 100 or 150 MHz). Chemical shifts *δ* are in ppm, and the following abbreviations are used: singlet (s), doublet (d), triplet (t), multiplet (m), broad singlet (brs). Mass spectra (MS) were determined on an Agilent 1100-sl mass spectrometer (ESI) from Agilent Co., Ltd. High resolution accurate mass determinations (HRMS) were run on a Bruker Micromass Time of Flight mass spectrometer (ESI). The IR spectra were obtained on a Bruker IFS 55 FT-IR spectrophotometer using KBr disks. Melting points were measured by an X-4 Micro-Melting point detector (Beijing Tech Instrument Co., Ltd.), without correction.

### General experimental section

#### General procedure for synthesis of 3-arylselenylindoles and 3-arylthioindoles

Method A: Based on the reported method^12^, a mixture of the appropriate indole (0.6 mmol), 1,2-bis(3-(allyloxy)-4-methoxyphenyl)diselenide (**8a**) or 1,2-bis(3- (allyloxy)-4-methoxyphenyl) disulfide (**8b**) (0.35 mmol), FeCl_3_ (20 mol%) and I_2_ (1 mol%, 0.0001 g/mL in CH_3_CN) was placed in a 25 mL vessel. The reaction mixture was irradiated in the water bath of an ultrasonic cleaner at room temperature for 9 h. After the evaporation of the solvent, the residual crude product was purified by preparative thin-layer chromatography with *n*-hexane-AcOEt (v/v = 5:1) or pure CH_2_Cl_2_ and analyzed by MS, HRMS, ^1^H-NMR, ^13^C-NMR and IR. See the supplementary information for the characterization details.

Method B: Similar to method A, a mixture of the appropriate indole (0.6 mmol), 1,2-bis(3-(allyloxy)-4-methoxyphenyl)diselenide (**8a**) or 1,2-bis(3-(allyloxy)- 4-methoxyphenyl) disulfide (**8b**) (0.35 mmol), FeCl_3_ (20 mol%) and I_2_ (1 mol%, 0.0001 g/mL in CH_3_CN) was placed in a 25 mL vessel. The reaction mixture was refluxed at 80 °C for 20 h in an oil bath. After the evaporation of the solvent, the residual crude product was purified by preparative thin-layer chromatography with *n*-hexane-AcOEt (v/v = 5:1) or pure CH_2_Cl_2_.

#### General procedure for synthesis of 3′-hydroxyl-3-arylselenylindoles and 3′-hydroxyl-3-arylthioindoles

Method A: Based on the reported method^36^, allyl ether (0.2 mmol) dissolved in anhydrous THF (10 mL), Pd(PPh_3_)_4_ (0.02 mmol) and NaBH_4_ (0.06 g, 1.6 mmol) were added under a nitrogen atmosphere. The reaction mixture was placed in a water bath of an ultrasonic cleaner and irradiated for 3 h. The reaction mixture was filtered and the solvent was evaporated to leave a residue which was purified by preparative thin-layer chromatography with *n*-hexane-AcOEt (v/v = 4:1) and analyzed by MS, HRMS, ^1^H-NMR, ^13^C-NMR and IR. See the supplementary information for the characterization details.

Method B: Similar to method A, to a solution of allyl ether (0.2 mmol) in anhydrous THF (10 mL) under a nitrogen atmosphere, Pd(PPh_3_)_4_ (0.02 mmol) and NaBH_4_ (0.06 g, 1.6 mmol) were added. The reaction mixture was stirred at 25 °C for 15 h. The reaction mixture was filtered and the solvent was evaporated to leave a residue which was purified by preparative thin-layer chromatography with *n*-hexane-AcOEt (v/v = 4:1).

#### Antiproliferative activity assay

The *in vitro* antiproliferative activity assay was performed following a previously reported method^12^.

#### Immunofluorescence studies

Immunofluorescence studies were performed following a previously reported method with 14 nM of **10a**, or 11 nM of CA-4^12^.

## Additional Information

**How to cite this article**: Wen, Z. *et al.* Ultrasound-promoted two-step synthesis of 3-arylselenylindoles and 3-arylthioindoles as novel combretastatin A-4 analogues. *Sci. Rep.* **6**, 23986; doi: 10.1038/srep23986 (2016).

## Supplementary Material

Supplementary Information

## Figures and Tables

**Figure 1:**
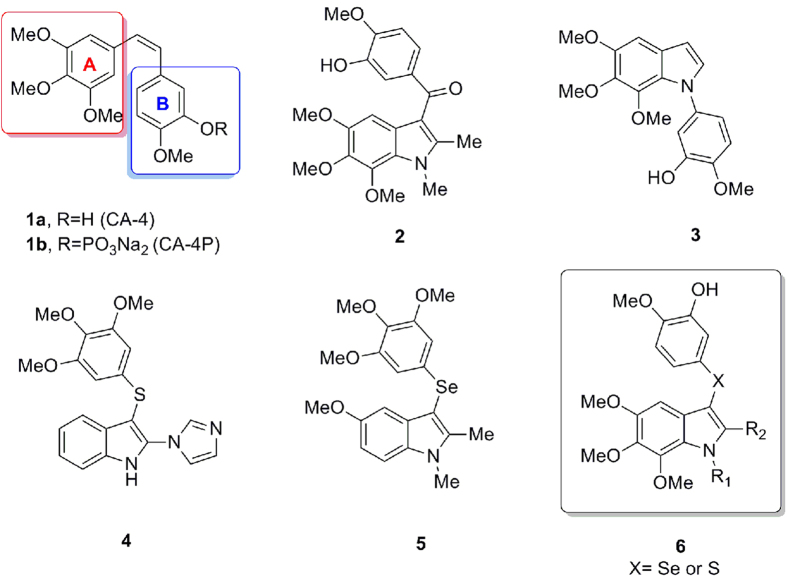
The structures of CA-4 (**1a**), CA-4P (**1b**), the analogues (**2**–**5**) and the general structure of target compounds (**6**).

**Table 1:**
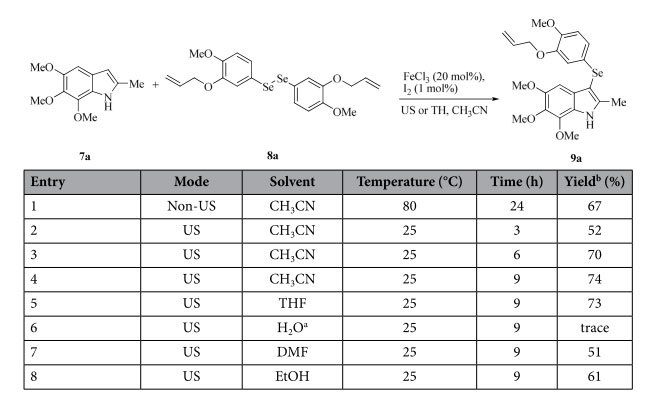
The optimization of conditions for the preparation of compound **9a**.

^a^Fe_2_(SO_4_)_3_.5H_2_O (20 mol%) was used instead of FeCl_3_.

^b^isolated yield.

**Table 2:**
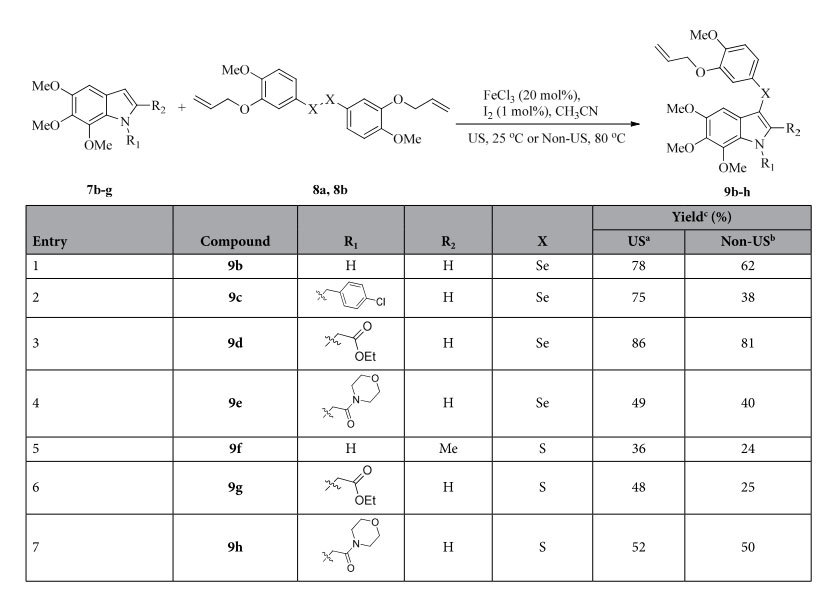
Synthesis of compounds 9b–h.

^a^US: 25 °C, 9 h.

^b^Non-US: 80 °C, 20 h.

^c^isolated yield.

**Table 3:**
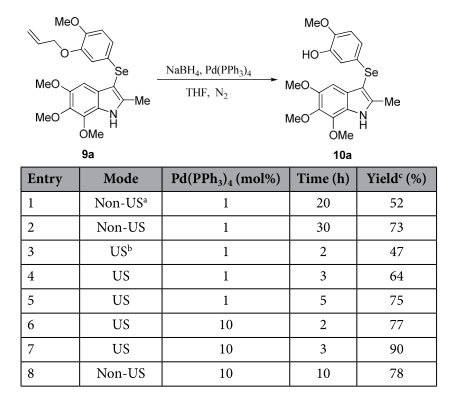
The optimization of conditions for the preparation of compound 10a.

^a^Non-US: 25 °C.

^b^US: 25 °C.

^c^isolated yield.

**Table 4:**
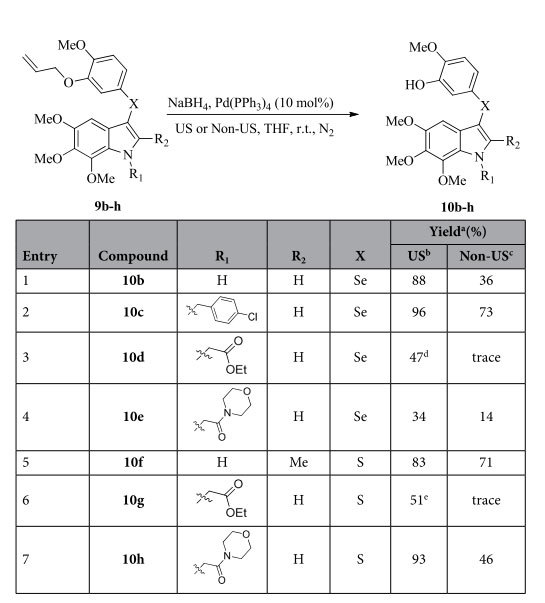
*O*-Deallylations of compounds 10b–h.

^a^isolated yield.

^b^US: 25 °C, 3 h.

^c^Non-US: 25 °C, 15 h.

^d^The highest yield (90%) was obtained under US irradiation at 25 °C for 1.3 h.

^e^The highest yield (65%) was obtained under US irradiation at 25 °C for 2 h.

**Table 5:** The *in vitro* antiproliferative activities of the synthesized compounds against three human cancer cell lines.

Compound	IC_50_^a^ (μM ± SD)		
	SGC7901	KB	HT1080
10a	0.054 ± 0.009^b^	0.025 ± 0.003^b^	0.014 ± 0.002^b^
10b	0.22 ± 0.03^b^	0.64 ± 0.05^b^	1.0 ± 0.2^b^
10c	14.92 ± 1.8^c^	7.82 ± 0.6^c^	12.55 ± 1.1^c^
10d	22.04 ± 1.5^c^	17.69 ± 1.1^c^	>500^c^
10e	10.56 ± 1.1^c^	11.72 ± 0.8^c^	18.68 ± 2.2^c^
10f	>500^c^	>500^c^	>500^c^
10g	>500^c^	278.4 ± 22^c^	>500^c^
10h	23.78 ± 1.2^c^	5.45 ± 0.8^c^	16.70 ± 1.2^c^
CA-4	0.011 ± 0.0012^c^	0.004 ± 0.0007^c^	0.011 ± 0.0018^c^

^a^IC_50_, expressed as the concentration of drug inhibiting cell growth by 50%. Data are expressed as means ± SDs (standard deviations).

^b^n = 4.

^c^n = 3.
