# Supplementary material for: Ultrasound-promoted two-step synthesis of 3-arylselenylindoles and 3-arylthioindoles as novel combretastatin A-4 analogues
Source: Sci Rep. 2016 Apr 5;6:23986. doi: 10.1038/srep23986 (PMC4820744; doi:10.1038/srep23986)

Supplementary Information

Ultrasound-promoted two-step synthesis of 3-arylselenylindoles and 3-arylthioindoles as novel combretastatin A-4 analogues

Zhiyong Wen[1], Xiaona Li[1], Daiying Zuo[2], Binyue Lang[2], Yang Wu[1], Mingyang Jiang[1], Huizhuo Ma[1], Kai Bao[1, 3,] *, Yingliang Wu[2], Weige Zhang[1,]*

[1] Key Laboratory of Structure-Based Drug Design and Discovery, Ministry of Education, Shenyang Pharmaceutical University, 103 Wenhua Road, Shenhe District, Shenyang 110016 (China)

[2] Department of Pharmacology, Shenyang Pharmaceutical University, 103 Wenhua Road, Shenhe District, Shenyang 110016 (China)

[3] Division of Hematology/ Oncology, Department of Medicine, Beth Israel Deaconess Medical Center and Harvard Medical School, Boston, MA 02215 (USA)

* **Corresponding Authors:** Weige Zhang, Ph.D.

103 Wenhua Road. Room 105 Huagong building

Shenhe District, Shenyang, China, 110016

Office/Fax: 86-24-23986422;

E-mail: zhangweige2000@sina.com

Kai Bao, Ph.D.

419 Room 330 Brookine Avenue, Boston, MA, US, 02215

Office/Fax: *+1 617 6670678*;

E-mail: kbao@bidmc.harvard.edu

List of content

1. **Synthesis of compound 7a-7e, 8a and 8b** **S3-7**
2. **Synthesis of 3-arylselenylindoles and 3-arylthioindoles (9a-9h)** **S7-12**
3. **Synthesis of 3'-hydroxyl-3-arylselenylindoles and 3'-hydroxyl-3-aryl-thioindoles (10a-10h)** **S12-16**
4. **^{1}H NMR and ^{13}C NMR spectra of compound 7a-7e, 8a and 8b** **S17-24**
5. **^{1}H NMR and ^{13}C NMR spectra of products 9a-9h** **S25-32**
6. **^{1}H NMR and ^{13}C NMR spectra of products 10a-10h** **S33-40**

Synthesis of indole 7a-7e

5,6,7-Trimethoxy-2-methyl-1*H*-indole (**7a**)

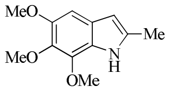

Step one: 3,4,5-Trimethoxybenzoic acid (20.0 g) suspended in MeOH (120 mL) and H_2SO_4 (10 mL) were refluxed for 7 h, and the completion of the reaction was confirmed by TLC. The reaction mixture was cooled to room temperature then diluted with H_2O (300 mL) and extracted with EtOAc (3×150 mL). The combined organic layers were washed with NaOH aq. (2 M, 2×100 mL) and brine, dried over anhydrous Na_2SO_4, filtered, and the solvent was removed *in vacuo*. The crude product was recrystallized with EtOH to yield methyl 3,4,5-trimethoxybenzoate as a white solid (18.1 g, 85%); m.p. 79-80 °C; ^{1}H NMR (400 MHz, $CDCl_3$): δ_H 3.91 (12H, s, 4×OCH_3), 7.30 (2H, s, 2×ArH). MS (ESI, pos) 227.1 $[M+H]^+$, 249.1 $[M+Na]^+$, 475.1 $[2M+Na]^+$.

Step two: Based on a literature method[24], 3,4,5-trimethoxybenzoate (18.1 g, 80 mmol) was dissolved in Ac_2O (200 mL) with stirring, and $(NH_4)_2[Ce(NO_3)_6]$ (21.93 g, 40 mmol) was then added in portions to this solution at 60 °C. The mixture was stirred for another 2 h until complete consumption of the starting material (confirmed by TLC). The reaction mixture was poured into crushed ice, and pale yellow crystalline precipitated was filtered and washed with H_2O (3×30 mL). Methyl 3,4,5-trimethoxy-2-nitrobenzoate was obtained as a pale yellow solid (19.3 g, 89%), m.p. 61-63 °C; ^{1}H NMR (400 MHz, $CDCl_3$): δ_H 3.88 (3H, s, OCH_3), 3.96 (6H, s, 2×OCH_3), 3.97 (3H, s, OCH_3), 7.28 (1H, s, ArH). MS (ESI, pos) 272.1 $[M+H]^+$, 294.1 $[M+Na]^+$, 310.1 $[M+K]^+$, 564.6 $[2M+Na]^+$.

Step three: Following a previously reported method[25], sodium borohydride (13.82 g, 365.3 mmol) was added to methyl 3,4,5-trimethoxy-2-nitrobenzoate (19.3 g, 71.16 mmol) in H_2O /dioxane (200 mL, v/v = 1:1) at room temperature with stirring for 5 h, and the completion of the reaction was confirmed by TLC. The reaction was quenched by HCl aq. (1 M) and extracted with EtOAc (3×100 mL). The combined organic layers were washed with brine, dried over Na_2SO_4 and concentrated *in vacuo*. The residue was purified by flash chromatography on silica gel (200-300 mesh) with *n*-hexane-EtOAc (v/v = 4:1) to afford (3,4,5-trimethoxy-2-nitrophenyl)methanol as a pale yellow solid (8.6 g, 50%), m.p. 68-69 °C; ^{1}H NMR (400 MHz, $CDCl_3$): δ_H 2.25 (1H, brs, OH), 3.89 (3H, s, OCH_3), 3.94 (3H, s, OCH_3), 3.99 (3H, s, OCH_3), 4.63 (2H, s, OCH_2), 6.84 (1H, s, ArH).

MS (ESI, pos) 266.1 $[M+Na]^+$, 282.3 $[M+K]^+$, 508.8 $[2M+Na]^+$.

Step four: Following a previously reported method[22], PCC (11.43 g, 53.0 mmol) was added to (3,4,5-trimethoxy-2-nitrophenyl)methanol (8.6 g, 35.36 mmol) in CH_2Cl_2 (50 mL) at room temperature with stirring. The reaction mixture was stirred for 1 h, and concentrated *in vacuo* and Et_2O (150 mL) was added in, then the resuting mixture filtered through *Celite*, and the *Celite* was washed with Et_2O (3×50 mL). The filtrate was evaporated *in vacuo*, and the residue was purified by column chromatography on silica gel (200-300 mesh) with *n*-hexane-EtOAc (v/v = 4:1) to afford 3,4,5-trimethoxy-2-nitrobenzaldehyde as a pale yellow solid (4.1 g, 48%), m.p. 69-71 °C; ^{1}H NMR (400 MHz, $CDCl_3$): δ_H 3.98 (3H, s, OCH_3), 4.00 (6H, s, 2×OCH_3), 7.23 (1H, s, ArH), 9.88 (1H, s, CHO). MS (ESI, pos) 242.1 $[M+H]^+$, 264.1 $[M+Na]^+$.

Step five: Following a previously reported method[23], 3,4,5-trimethoxy-2-nitrobenzaldehyde (4.1 g, 17 mmol) was used to furnish 1,2,3-trimethoxy-4-nitro-5-(2-nitroprop-1-enyl)benzene as a pale yellow solid (4.50 g, 85%), m.p. 111-112 °C; ^{1}H NMR (400 MHz, $CDCl_3$): δ_H 2.32 (3H, s, CH_3), 3.94 (3H, s, OCH_3), 3.95 (3H, s, OCH_3), 4.02 (3H, s, OCH_3), 6.56 (1H, s, ArH), 7.90 (1H, s, =CH). MS (ESI, pos) 299.2 $[M+H]^+$, 321.1 $[M+Na]^+$, 337.0 $[M+K]^+$.

Step six: Following a previously reported method [27], 1,2,3-trimethoxy-4-nitro-5-(2-nitroprop-1-enyl)benzene (4.50 g) was used to afford compound **7a** as an off-white solid (2.51 g, 75%), m.p. 97-99 °C; ^{1}H NMR (400 MHz, $CDCl_3$): δ_H 2.41 (3H, s, CH_3), 3.88 (3H, s, OCH_3), 3.89 (3H, s, OCH_3), 4.05 (3H, s, OCH_3), 6.09 (1H, brs, ArH), 6.74 (1H, s, ArH), 7.99 (1H, brs, NH); ^{13}C NMR (100 MHz, $CDCl_3$): δ_C 13.7, 56.5, 61.0, 61.5, 96.9, 100.4, 124.2, 124.7, 134.9, 137.2, 138.3,148.9. MS (ESI, pos) 222.1 $[M+H]^+$, 244.1 $[M+Na]^+$, 465.1 $[2M+Na]^+$.

5,6,7-Trimethoxy-1*H*-indole (**7b**)

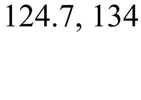

Following the procedure similar to that for 1,2,3-trimethoxy-4-nitro-5-(2-nitroprop-1-enyl)benzene, nitromethane (60 mL) and 3,4,5-trimethoxy-2-nitrobenzaldehyde (4.1 g) were used to give (Z)-1,2,3-trimethoxy-4-nitro-5-(2-nitrovinyl)benzene as a pale yellow solid (2.85 g, 57%), m.p. 158-160 °C; ^{1}H NMR (400 MHz, $CDCl_3$): δ_H 3.97 (6H, s, 2×OCH_3), 4.00 (3H, s, OCH_3), 6.79 (1H,

s, ArH), 7.45 (1H, d, J 13.2 Hz, CH=), 7.89 (1H, d, J 13.2 Hz, CH=). MS (ESI, pos) 285.1 $[M+H]^+$, 307.1 $[M+Na]^+$. Compound **7b** was obtained from (Z)-1,2,3-trimethoxy-4-nitro-5-(2-nitrovinyl)benzene (2.85 g) using the similar method to **7a** as an off-white solid (1.87 g, 79%), m.p. 92-94 °C; ^{1}H NMR (400 MHz, $CDCl_3$): δ_H 3.90 (3H, s, OCH_3), 3.91 (3H, s, OCH_3), 4.07 (3H, s, OCH_3), 6.44 (1H, dd, J 2.7, 2.5 Hz, ArH), 6.85 (1H, s, ArH), 7.12 (1H, t, J 2.7 Hz, ArH), 8.22 (1H, brs, NH). MS (ESI, pos) 208.1 $[M+H]^+$, 230.1 $[M+Na]^+$.

1-(4-Chlorobenzyl)-5,6,7-trimethoxy-1*H*-indole (**7c**)

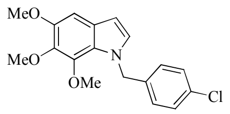

To a stirred solution of compound **7b** (0.4 g, 1.94 mmol) in acetone (10 mL) was slowly added potassium *tert*-butanolate (0.33 g, 2.91 mmol) and 1-chloro-4-(chloromethyl)benzene (0.38 g, 2.35 mmol) at room temperature. After 2 h, the reaction mixture was diluted with water (30 mL), and extracted with CH_2Cl_2 (3×30 mL). The combined organic layers were washed with brine, dried over Na_2SO_4 and concentrated *in vacuo*. The crude product was purified by preparative thin-layer chromatography with *n*-hexane-AcOEt (v/v = 5:1) to afford **7c** as a colourless oil (0.40 g, 63%). ^{1}H NMR (400 MHz, $CDCl_3$): δ_H 3.69 (3H, s, OCH_3), 3.86 (3H, s, OCH_3), 3.89 (3H, s, OCH_3), 5.50 (2H, s, NCH_2), 6.42 (1H, d, J 3.0 Hz, ArH), 6.84 (1H, s, ArH), 6.96 (3H, m, 3×ArH), 7.24 (2H, d, J 8.7 Hz, 2×ArH). MS (ESI, pos) 332.2 $[M+H]^+$, 354.1 $[M+Na]^+$.

Ethyl 2-(5,6,7-trimethoxy-1*H*-indol-1-yl)acetate (**7d**)

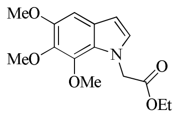

To a stirred solution of compound **7b** (0.4 g, 1.94 mmol) in anhydrous DMF (10 mL) at 0 °C was added NaH (0.24 g, 9.66 mmol) in portions. After 1 h, to the reaction mixture was added ethyl 2-bromoacetate (0.48 g, 2.96 mmol). The reaction accomplished within 20 min, then, the mixture was quenched with EtOH (3 mL), diluted with water and extracted with CH_2Cl_2 (3×30 mL). The combined organic layers were washed with brine, dried over Na_2SO_4 and concentrated *in vacuo*. The crude product was purified by preparative thin-layer chromatography with *n*-hexane-AcOEt

(v/v = 5:1) to afford **7d** as a colourless oil (0.28 g, 48%). ^{1}H NMR (400 MHz, $CDCl_3$): δ_H 1.26 (3H, t, *J* 6.6 Hz, CH_3), 3.87 (6H, s, 2×OCH_3), 3.95 (3 H, s, OCH_3), 4.22 (2H, q, *J* 6.6 Hz, OCH_2), 4.99 (2H, s, NCH_2), 6.38 (1H, s, ArH), 6.81 (1H, s, ArH), 6.88 (1H, s, ArH). MS (ESI, pos) 294.1 $[M+H]^+$, 316.1 $[M+Na]^+$.

1-Morpholino-2-(5,6,7-trimethoxy-1*H*-indol-1-yl)ethanone (**7e**)

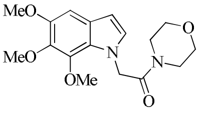

To a stirred solution of compound **7d** (0.57 g, 1.93 mmol) in MeOH (20 mL) at room temperature, a solution of KOH (0.22 g, 3.86 mmol) in H_2O (4 mL) was added. After 4 h, the reaction mixture was diluted with water (30 mL), and washed with CH_2Cl_2 (2×30 mL). The aqueous phase obtained was acidified with HCl (18% aqueous), and extracted with CH_2Cl_2 (3×30 mL). The combined organic layers were washed with brine, dried over Na_2SO_4 and concentrated *in vacuo*. The residue was dissolved in dry CH_2Cl_2 (5 mL), DCC (0.48 g, 2.32 mmol), morpholine (0.18 g, 2.12 mmol) and DMAP (0.18 g, 1.45 mmol) were added. The mixture was stirred under a N_2 atmosphere overnight at room temperature. After filtration, the filtrate was evaporated to dryness *in vacuo*, and the crude product was purified by preparative thin-layer chromatography with *n*-hexane-AcOEt-formic acid (v/v/v = 1:1:0.005) to afford **7e** as a white solid (0.45 g, 69% for two steps), m.p. 125-126 °C, ^{1}H NMR (400 MHz, $CDCl_3$): δ_H 3.53 (2H, m, morpholine H), 3.69 (6H, m, morpholine H), 3.88 (6H, s, 2×OCH_3), 3.95 (3H, s, OCH_3), 5.15 (2H, s, NCH_2), 6.41 (1H, d, *J* 3.1 Hz, ArH), 6.82 (1H, s, ArH), 6.92 (1H, d, *J* 3.1 Hz, ArH). MS (ESI, pos) 335.2 $[M+H]^+$, 357.2 $[M+Na]^+$, 373.1 $[M+K]^+$.

1,2-Bis(3-(allyloxy)-4-methoxyphenyl)diselenide (**8a**)

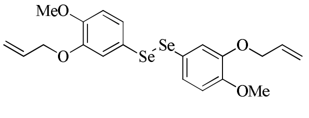

Following the previously reported methods[12], 1,2-Bis(3-(allyloxy)-4-methoxyphenyl) diselenide (**8a**) was obtained from 3-(allyloxy)-4-methoxyaniline (5.87 g, 32.75 mmol) as a mixture of 1,2-bis(3-(allyloxy)-4-methoxyphenyl)diselenide with 1,1'-selenobis(3-(allyloxy)-4-

methoxybenzene) (1:2.16, HPLC), as a yellow solid (3.0 g), which was used without further separation. ^{1}H NMR (400 MHz, $CDCl_3$) δ_H 3.86 (6H, s, 2×OCH_3), 4.53 (4H, d, *J* 5.5 Hz, 2×$ArOCH_2$), 5.27 (2H, d, *J* 10.6 Hz, 2×CH=), 5.37 (2H, d, *J* 16.1 Hz, 2×CH=), 6.03 (2H, m, 2×CH=), 6.76 (2H, d, *J* 8.3 Hz, 2×ArH), 7.10 (2H, d, *J* 1.3 Hz, 2×ArH), 7.15 (2H, dd, *J* 8.3, 1.3 Hz, 2×ArH); ^{13}C NMR (100 MHz, $CDCl_3$): δ_C 56.0 (2×C), 69.9 (2×C), 111.8 (2×C), 118.4 (2×C), 118.8 (2×C), 121.8 (2×C), 126.7 (2×C), 132.8 (2×C), 148.1 (2×C), 150.1 (2×C). HRMS (ESI): calc. for $C_{20}H_{22}NaO_4Se_2$ $[M+Na]^+$ 508.9741, found 508.9744.

1,2-Bis(3-(allyloxy)-4-methoxyphenyl) disulphide (**8b**)

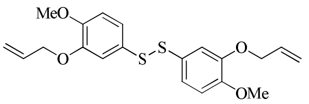

Following the previously reported methods[28-29], 3-(allyloxy)-4-methoxyaniline (4.0 g, 22.32 mmol) was used to furnish the crude 3-(allyloxy)-4-methoxybenzenethiol, which was used without further purification. To the obtained benzenethiol were added DMF (20 mL) and Et_3N (2 g, 20 mmol). Then, it was placed in an ultrasonic bath at room temperature and radiated for 1 h, The reaction mixture was extracted with CH_2Cl_2 (3×50 mL), while the combined organic layers were washed with water (3×30 mL) to eliminate the DMF entirely. The solvent was removed under vacuum to afford the crude product, which was purified by column chromatography on silica gel (200-300 mesh) with *n*-hexane-EtOAc (v/v = 10:1) to afford **8b** as an off-white solid (0.96 g, 22% for 4 steps), m.p. 77-79 °C; ^{1}H NMR (400 MHz, $CDCl_3$): δ_H 3.86 (6H, s, 2×OCH_3), 4.54 (4H, d, *J* 5.5 Hz, 2×$ArOCH_2$), 5.27 (2H, dd, *J* 10.4, 1.3 Hz, 2×CH=), 5.36 (2H, dd, *J* 17.4, 1.3 Hz, 2×CH=), 6.03 (2H, m, 2×CH=), 6.79 (2H, d, *J* 8.1 Hz, 2×ArH), 7.02 (2H, s, 2×ArH), 7.03 (2H, dd, *J* 8.1, 2.1 Hz, 2×ArH); ^{13}C NMR (100 MHz, $CDCl_3$): δ_C 56.0 (2×C), 69.9 (2×C), 111.7 (2×C), 115.8 (2×C), 118.3 (2×C), 123.9 (2×C), 128.5 (2×C), 132.8 (2×C), 148.2 (2×C), 149.9 (2×C). MS (ESI, pos) 413.2 $[M+Na]^+$. HRMS (ESI): calc. for $C_{20}H_{22}NaO_4S_2$ $[M+Na]^+$ 413.0852, found 413.0855.

Synthesis of 3-arylselenylindoles and 3-arylthioindoles

3-((3-(Allyloxy)-4-methoxyphenyl)selenyl)-5,6,7-trimethoxy-2-methyl-1*H*-indole (**9a**, Table 1)

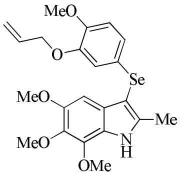

Using method A, compound **9a** was obtained from 5,6,7-trimethoxy-2-methyl-1*H*-indole (**7a**) and diselenide **8a** as a pale yellow oil which will slowly solidified upon standing (0.205 g, 74%), ^{1}H NMR (400 MHz, $CDCl_3$): δ_H 2.52 (3H, s, CH_3), 3.79 (3H, s, OCH_3), 3.85 (3H, s, OCH_3), 3.89 (3H, s, OCH_3), 4.07 (3H, s, OCH_3), 4.43 (2H, td, *J* 5.5, 1.27 Hz, $ArOCH_2$), 5.13 (1H, dd, *J* 2.9, 1.5 Hz, CH=), 5.16 (1H, m, CH=), 5.91 (1H, m, OCH_2CH=), 6.69 (1H, d, *J* 8.3Hz, ArH), 6.77 (3H, m, 3×ArH), 8.30 (1H, brs, NH); ^{13}C NMR (100 MHz, $CDCl_3$): δ_C 13.3, 56.2, 56.4, 61.1, 61.4, 69.8, 96.6, 97.2, 112.5, 114.6, 118.0, 121.5, 123.4, 124.1, 127.1, 133.1, 137.7, 138.3, 140.1, 147.9, 148.3, 149.8. MS (ESI, pos) 464.1 $[M+H]^+$, 486.1 $[M+Na]^+$. HRMS (ESI): calc. for $C_{22}H_{26}NO_5Se$ $[M+H]^+$ 464.0971, found 464.0997. IR (KBr, *v* cm^{-1}): 3377, 2937, 2843, 1631, 1581, 1496, 1462, 1385, 1052, 1032, 1013, 876.

3-(3-(Allyloxy)-4-methoxyphenylselenyl)-5,6,7-trimethoxy-1*H*-indole (**9b**, Table 2, entry 1)

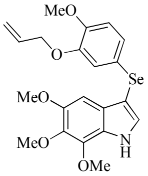

Using method A, compound **9b** was obtained from 5,6,7-trimethoxy-1*H*-indole (**7b**) and diselenide **8a** as a pale yellow oil which will slowly solidified upon standing (0.21 g, 78%), ^{1}H NMR (400 MHz, $CDCl_3$): δ_H 3.79 (3H, s, OCH_3), 3.86 (3H, s, OCH_3), 3.90 (3H, s, OCH_3), 4.08 (3H, s, OCH_3), 4.44 (2H, td, *J* 5.5, 1.4 Hz, $ArOCH_2$), 5.12 (1H, m, CH=), 5.16 (1H, m, CH=), 5.90 (1H, m, CH=), 6.70 (1H, d, *J* 8.3 Hz, ArH), 6.80 (1H, s, ArH), 6.83 (1H, d, *J* 2.0 Hz, ArH), 6.86 (1H, dd, *J* 8.3, 2.0 Hz, ArH), 7.37 (1H, d, *J* 2.5 Hz, ArH), 8.48 (1H, brs, NH); ^{13}C NMR (100 MHz, $CDCl_3$): δ_C 56.0 (2×C), 69.8, 69.9 (2×C), 111.8, 112.3, 118.3 (2×C), 118.4, 118.8, 121.8, 121.9, 126.1, 126.7, 132.8, 132.9, 148.0, 148.3, 149.2, 150.0. MS (ESI, pos) 450.1 $[M+H]^+$, 472.0 $[M+Na]^+$, 921.2 $[2M+Na]^+$. HRMS (ESI): calc. for $C_{21}H_{24}NO_5Se$ $[M+H]^+$ 450.0814, found 450.0836. IR (KBr, *v* cm^{-1}): 3346, 2917, 2848, 1580, 1496, 1463, 1248, 1135, 876.

3-(3-(Allyloxy)-4-methoxyphenylselenyl)-1-(4-chlorobenzyl)-5,6,7-trimethoxy-1*H*-indole (**9c**, Table 2, entry 2)

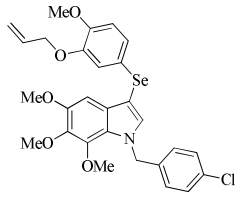

Using method A, compound **9c** was obtained from 1-(4-chlorobenzyl)-5,6,7-trimethoxy-1*H*-indole (**7c**) and diselenide **8a** as a white solid (0.258 g, 75%), m.p. 99-100 °C, ^{1}H NMR (400 MHz, $CDCl_3$): δ_H 3.72 (3H, s, OCH_3), 3.80 (3H, s, OCH_3), 3.85 (3H, s, OCH_3), 3.85 (3H, s, OCH_3), 4.42 (2H, td, J= 5.6, 1.5 Hz, $ArOCH_2$), 5.13 (1H, m, CH=), 5.16 (1H, m, CH=), 5.52 (2H, s, NCH_2Ar), 5.90 (1H, m, CH=), 6.71 (1H, d, *J* 8.5 Hz, ArH), 6.81 (2H, m, 2×ArH), 6.86 (1H, dd, *J* 8.3, 2.0 Hz, ArH), 7.00 (2H, d, *J* 8.3 Hz, 2×ArH), 7.26 (3H, m, 3×ArH); ^{13}C NMR (100 MHz, $CDCl_3$): δ_C 29.7, 51.5, 56.0, 56.2, 61.2, 61.3, 69.8, 96.9, 98.1, 112.4, 115.0, 118.1, 122.0, 123.8, 123.9, 127.7 (2×C), 128.8 (2×C), 133.0, 133.2, 135.4, 137.2, 139.3, 140.4, 148.1, 148.3, 150.0. HRMS (ESI): calc. for $C_{28}H_{28}ClNNaO_5Se$ $[M+Na]^+$ 596.0713, found 596.0705.

Ethyl 2-(3-(3-(allyloxy)-4-methoxyphenylselenyl)-5,6,7-trimethoxy-1*H*-indol-1-yl)acetate (**9d**, Table 2, entry 3)

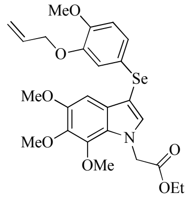

Using method A, compound **9d** was obtained from ethyl 2-(5,6,7-trimethoxy-1*H*-indol-1-yl)acetate (**7d**) and diselenide **8a** as a white solid (0.256 g, 86%), m.p. 63-64 °C, ^{1}H NMR (400 MHz, $CDCl_3$): δ_H 1.28 (3H, t, *J* 7.2 Hz, CH_3), 3.79 (3H, s, OCH_3), 3.84 (3H, s, OCH_3), 3.87 (3H, s, OCH_3), 3.97 (3H, s, OCH_3), 4.24 (2H, q, *J* 7.2 Hz, OCH_2), 4.45 (2H, d, *J* 5.4 Hz, $ArOCH_2$), 5.03 (2H, s, NCH_2), 5.14 (2H, m, CH_2=), 5.90 (1H, m, CH=), 6.70 (1H, d, *J* 8.3 Hz, ArH), 6.79 (1H, s, ArH), 6.80 (1H, d, *J* 2.0 Hz, ArH), 6.85 (1H, dd, *J* 8.3, 2.0 Hz, ArH), 7.15 (1H, s, ArH); ^{13}C NMR (100 MHz, $CDCl_3$): δ_C 14.2, 50.1, 56.0, 56.3, 61.2, 61.3, 61.4, 69.7,

97.0, 98.1, 112.4, 114.7, 118.0, 121.8, 123.9, 124.3, 127.5, 133.1, 135.9, 139.1, 140.2, 148.0, 148.3, 150.0, 168.9. MS (ESI, pos) 536.1 $[M+H]^+$, 558.1 $[M+Na]^+$, 574.1 $[M+K]^+$, MS (ESI, neg) 533.9 $[M-H]^-$. HRMS (ESI): calc. for $C_{25}H_{30}NO_7Se$ $[M+H]^+$ 536.1182, found 536.1196. IR (KBr, ν cm^{-1}): 3427, 2925, 1739, 1638, 1490, 1384, 874.

2-(3-(3-(Allyloxy)-4-methoxyphenylselenyl)-5,6,7-trimethoxy-1*H*-indol-1-yl)-1-morpholinoethanone (**9e**, Table 2, entry 4)

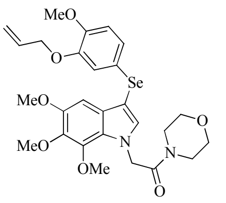

Using method A, compound **9e** was obtained from 1-morpholino-2-(5,6,7-trimethoxy-1*H*-indol-1-yl)ethanone (**7e**) and diselenide **8a** as a white solid (0.169 g, 49%), m.p. 126-128 °C, ^{1}H NMR (400 MHz, $CDCl_3$): δ_H 3.53 (2H, m, morpholine H), 3.65 (2H, m, morpholine H), 3.72 (4H, m, morpholine H), 3.79 (3H, s, OCH_3), 3.83 (3H, s, OCH_3), 3.87 (3H, s, OCH_3), 3.95 (3H, s, OCH_3), 4.46 (2H, d, *J* 5.6 Hz, $ArOCH_2$), 5.15 (2H, s, NCH_2), 5.16 (2H, m, CH_2=), 5.91 (1H, m, CH=), 6.70 (1H, d, *J* 6.3 Hz, ArH), 6.80 (1H, s, ArH), 6.82 (1H, d, *J* 1.8 Hz, ArH), 6.85 (1H, dd, *J* 7.0, 1.8 Hz, ArH), 7.17 (1H, s, ArH); ^{13}C NMR (100 MHz, $CDCl_3$): δ_C 42.2, 45.1, 49.5, 55.9, 56.2, 61.2, 61.3, 66.4, 66.8, 69.7, 97.2, 97.9, 112.4, 114.7, 117.9, 121.7, 123.9, 124.3, 127.4, 133.0, 136.1, 139.0, 139.9, 147.9, 148.2, 149.7, 166.3. MS (ESI, pos) 577.1 $[M+H]^+$, 599.2 $[M+Na]^+$, MS (ESI, neg) 574.9 $[M-H]^-$. HRMS (ESI): calc. for $C_{27}H_{32}N_2NaO_7Se$ $[M+Na]^+$ 599.1267, found 599.1324.

3-(3-(Allyloxy)-4-methoxyphenylthio)-5,6,7-trimethoxy-2-methyl-1*H*-indole (**9f**, Table 2, entry 6)

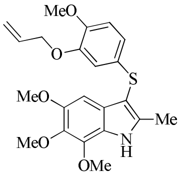

Using method A, compound **9f** was obtained from 5,6,7-trimethoxy-2-methyl-1*H*-indole (**7a**) and disulphide **8b** as a pael yellow oil (0.089 g, 36%) which will slowly solidified upon standing, ^{1}H NMR (400 MHz, $CDCl_3$): δ_H 2.47 (3H, s, CH_3), 3.79 (3H, s, OCH_3), 3.84 (3H, s, OCH_3), 3.89

(3H, s, OCH_3), 4.07 (3H, s, OCH_3), 4.45 (2H, d, *J* 5.4 Hz, $ArOCH_2$), 5.16 (2H, m, CH_2=), 5.93 (1H, m, CH=), 6.62 (1H, dd, *J* 8.3, 1.8 Hz, ArH), 6.67 (1H, d, *J* 1.8 Hz, ArH), 6.71 (1H, d, *J* 8.5 Hz, ArH), 6.76 (1H, s, ArH), 8.38 (1H, brs, NH); ^{13}C NMR (100 MHz, $CDCl_3$): δ_C 12.2, 56.1, 56.4, 61.1, 61.5, 69.9, 95.8, 100.4, 112.1, 112.4, 118.0, 118.6, 123.1, 126.3, 130.5, 133.2, 137.8, 138.4, 140.4, 147.3, 148.3, 149.9. HRMS (ESI): calc. for $C_{22}H_{25}NNaO_5S$ $[M+Na]^+$ 438.1346, found 438.1346. IR (KBr, *v* cm^{-1}): 3340, 2935, 1583, 1498, 1463, 1249, 1215, 802.

Ethyl 2-(3-(3-(allyloxy)-4-methoxyphenylthio)-5,6,7-trimethoxy-1*H*-indol-1-yl)acetate (**9g**, Table 2, entry 7)

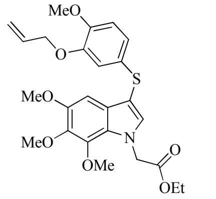

Using method A, compound **9g** was obtained from ethyl 2-(5,6,7-trimethoxy-1*H*-indol-1-yl)acetate (**7d**) and disulphide **8b** as a colorless oil (0.14 g, 48%), ^{1}H NMR (400 MHz, $CDCl_3$): δ_H 1.28 (3H, t, *J* 7.0 Hz, CH_3), 3.80 (3H, s, OCH_3), 3.82 (3H, s, OCH_3), 3.87 (3H, s, OCH_3), 3.97 (3H, s, OCH_3), 4.24 (2H, q, *J* 7.0 Hz, OCH_2), 4.47 (2H, d, *J* 5.5 Hz, $ArOCH_2$), 5.02 (2H, s, NCH_2), 5.15 (2H, m, CH_2=), 5.92 (1H, m, CH=), 6.71 (3H, m, 3×ArH), 6.77 (1H, s, ArH), 7.15 (1H, s, ArH); ^{13}C NMR (100 MHz, $CDCl_3$): δ_C 14.2, 50.1, 56.1, 56.2, 61.2, 61.3, 61.5, 69.8, 96.1, 102.9, 112.3, 118.0, 119.0, 124.4 (2×C), 126.7, 130.2, 133.1, 135.3, 139.2, 140.3, 147.4, 148.2, 150.1, 168.8. MS (ESI, pos) 488.2 $[M+H]^+$, 510.2 $[M+Na]^+$, 526.2 $[M+K]^+$.HRMS (ESI): calc. for $C_{25}H_{29}NNaO_7S$ $[M+Na]^+$ 510.1557, found 510.1569.

2-(3-(3-(Allyloxy)-4-methoxyphenylthio)-5,6,7-trimethoxy-1*H*-indol-1-yl)-1-morpholinoethanone (**9g**, Table 2, entry 9)

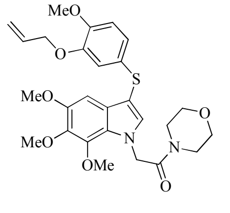

Using method A, compound **9h** was obtained from 1-morpholino-2-(5,6,7-trimethoxy-1*H*-indol-1-yl)ethanone (**7e**) and disulphide **8b** as a white solid (0.165 g, 52%), m.p.

129-130 °C, ^{1}H NMR (400 MHz, $CDCl_3$): δ_H 3.55 (2H, m, morpholine H), 3.66 (2H, m, morpholine H), 3.72 (4H, m, morpholine H), 3.79 (3H, s, OCH_3), 3.82 (3H, s, OCH_3), 3.87 (3H, s, OCH_3), 3.96 (3H, s, OCH_3), 4.48 (2H, d, J 5.4 Hz, $ArOCH_2$), 5.15 (2H, s, NCH_2), 5.18 (2H, m, CH_2=), 5.93 (1H, m, CH=), 6.72 (3H, m, 3×ArH), 6.78 (1H, s, ArH), 7.17 (1H, s, ArH); ^{13}C NMR (100 MHz, $CDCl_3$): δ_C 42.3, 45.2, 49.7, 56.1, 56.3, 61.2, 61.4, 66.5, 66.9, 69.8, 96.4, 102.7, 112.2, 112.3, 118.0, 118.9, 124.5, 126.7, 130.2, 133.1, 135.7, 139.2, 140.2, 147.3, 148.2, 149.9, 166.4. MS (ESI, pos) 529.2 $[M+H]^+$, 551.2 $[M+Na]^+$, 567.1 $[M+K]^+$, MS (ESI, neg) 527.0 $[M-H]^-$. HRMS (ESI): calc. for $C_{27}H_{32}N_2NaO_7S$ $[M+Na]^+$ 551.1822, found 551.1835.

Synthesis of 3'-hydroxyl-3-arylselenylindoles and 3'-hydroxyl-3-arylthioindoles

2-Methoxy-5-(5,6,7-trimethoxy-2-methyl-1*H*-indol-3-ylselenyl)phenol (**10a**, Table 3)

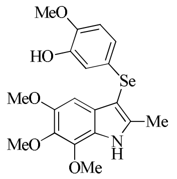

Using method A, compound **10a** was obtained from 3-(3-(allyloxy)-4-methoxyphenylselenyl)-5,6,7-trimethoxy-2-methyl-1*H*-indole (**9a**) as a pale yellow solid (0.076 g, 90%), m.p. 53-55 °C, ^{1}H NMR (400 MHz, $CDCl_3$): δ_H 2.50 (3H, s, CH_3), 3.79 (3H, s, OCH_3), 3.85 (3H, s, OCH_3), 3.89 (3H, s, OCH_3), 4.06 (3H, s, OCH_3), 5.59 (1H, brs, ArOH), 6.67 (1H, dd, J 7.5, 1.4 Hz, ArH), 6.76 (3H, m, 3×ArH), 8.40 (1H, brs, NH); ^{13}C NMR (100 MHz, $CDCl_3$): δ_C 13.2, 56.0, 56.4, 61.2, 61.4, 96.6, 97.0, 111.4, 115.3, 120.5, 123.4, 125.1, 127.2, 137.7, 138.3, 140.2, 145.1, 146.1, 149.7. MS (ESI, pos) 424.1 $[M+H]^+$, 869.0 $[2M+Na]^+$. HRMS (ESI): calc. for $C_{19}H_{21}NNaO_5Se$ $[M+Na]^+$ 446.0477, found 446.0478. IR (KBr, ν cm^{-1}): 3382, 3934, 1630, 1583, 1497, 1464, 1111, 911, 881.

2-Methoxy-5-(5,6,7-trimethoxy-1*H*-indol-3-ylselenyl)phenol (**10b**, Table 4, entry 1)

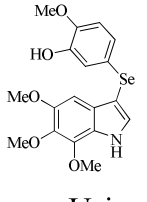

Using method A compound **10b** was obtained from 3-(3-(allyloxy)-4-methoxyphenylselenyl)-5,6,7-trimethoxy-1*H*-indole (**9b**) as a pale yellow solid

(0.072 g, 88%), m.p. 118-120 °C, ^{1}H NMR (400 MHz, $CDCl_3$): δ_H 3.81 (3H, s, OCH_3), 3.86 (3H, s, OCH_3), 3.90 (3H, s, OCH_3), 4.08 (3H, s, OCH_3), 5.55 (1H, brs, ArOH), 6.68 (1H, d, *J* 8.2 Hz, ArH), 6.81 (1H, s, ArH), 6.83 (2H, m, 2×ArH), 7.37 (1H, d, *J* 2.4 Hz, ArH), 8.48 (1H, brs, NH); ^{13}C NMR (150 MHz, $CDCl_3$): δ_C 55.9, 56.3, 61.1, 61.4, 96.5, 98.7, 111.3, 115.8, 121.0, 124.5, 124.8, 125.9, 130.6, 138.3, 138.7, 145.3, 146.0, 149.9. MS (ESI, pos) 410.0 $[M+H]^+$, 432.0 $[M+Na]^+$. HRMS (ESI): calc. for $C_{18}H_{19}NNaO_5Se$ $[M+Na]^+$ 432.0321, found 432.0320. IR (KBr, ν cm^{-1}): 3396, 2931, 1629, 1581, 1497, 1464, 1115, 877.

5-(1-(4-Chlorobenzyl)-5,6,7-trimethoxy-1*H*-indol-3-ylselenyl)-2-methoxyphenol (**10c**, Table 4, entry 2)

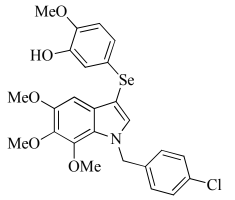

Using method A, compound **10c** was obtained from 3-(3-(allyloxy)-4-methoxyphenylselenyl)-1-(4-chlorobenzyl)-5,6,7-trimethoxy-1*H*-indole (**9c**) as a pale yellow solid (0.102 g, 96%), m.p. 145-147 °C, ^{1}H NMR (400 MHz, $CDCl_3$): δ_H 3.71 (3H, s, OCH_3), 3.82 (3H, s, OCH_3), 3.85 (6H, s, 2×OCH_3), 5.52 (2H, s, NCH_2), 5.57 (1H, brs, ArOH), 6.69 (1H, d, *J* 8.7 Hz, ArH), 6.82 (3H, m, 3×ArH), 6.99 (2H, d, *J* 8.5 Hz, 2×ArH), 7.22 (1H, s, ArH), 7.27 (2H, d, *J* 8.5 Hz, 2×ArH); ^{13}C NMR (100 MHz, $CDCl_3$): δ_C 51.5, 56.0, 56.2, 61.2, 61.3, 96.8, 97.9, 111.3, 115.7, 120.9, 123.9, 124.9, 127.7 (2×C), 127.8, 128.9 (2×C), 133.1, 135.7, 137.2, 139.2, 140.4, 145.3, 146.1, 149.9. MS (ESI, pos) 556.0 $[M+Na]^+$, MS (ESI, neg) 531.8 $[M-H]^-$. HRMS (ESI): calc. for $C_{25}H_{24}ClNNaO_5Se$ $[M+Na]^+$ 556.0400, found 556.0378. IR (KBr, ν cm^{-1}): 3348, 2929, 1618, 1574, 1496, 1465, 1441, 880.

Ethyl 2-(3-(3-hydroxy-4-methoxyphenylselenyl)-5,6,7-trimethoxy-1*H*-indol-1-yl)acetate (**10d**, Table 4, entry 3)

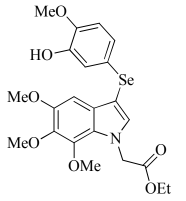

Using method A, compound **10d** was obtained from ethyl 2-(3-(3-(allyloxy)-4-methoxyphenylselenyl)-5,6,7-trimethoxy-1*H*-indol-1-yl)acetate (**9d**) as a pale yellow solid (0.047 g, 47%), m.p. 50-51 °C, ^{1}H NMR (400 MHz, $CDCl_3$): δ_H 1.27 (3H, t, *J* 7.2 Hz, CH_3), 3.80 (3H, s, OCH_3), 3.84 (3H, s, OCH_3), 3.87 (3H, s, OCH_3), 3.97 (3H, s, OCH_3), 4.24 (2H, q, *J* 7.2 Hz, OCH_2), 5.02 (2H, s, NCH_2), 5.57 (1H, brs, ArOH), 6.67 (1H, d, *J* 8.3 Hz, ArH), 6.82 (3H, m, 3×ArH), 7.15 (1H, s, ArH); ^{13}C NMR (100 MHz, $CDCl_3$): δ_C 14.2, 50.1, 56.0, 56.2, 61.1, 61.3, 61.4, 96.9, 98.0, 111.3, 115.6, 120.8, 124.2, 12.8, 127.5, 136.0, 140.0, 140.1, 145.2, 146.0, 149.9, 169.0. MS (ESI, pos) 496.1 $[M+H]^+$, 518.1 $[M+Na]^+$. HRMS (ESI): calc. for $C_{22}H_{25}NNaO_7Se$ $[M+Na]^+$ 518.0688, found 518.0708. IR (KBr, *v* cm^{-1}): 3419, 2918, 2848, 1750, 1621, 1578, 1496, 1465, 1439, 871.

2-(3-(3-Hydroxy-4-methoxyphenylselenyl)-5,6,7-trimethoxy-1*H*-indol-1-yl)-1-morpholinoethanone (**10e**, Table 4, entry 4)

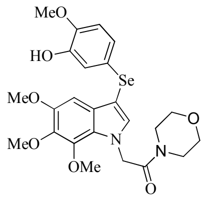

Using method A, compound **10e** was obtained from 2-(3-(3-(allyloxy)-4-methoxyphenylselenyl)-5,6,7-trimethoxy-1*H*-indol-1-yl)-1-morpholinoethanone (**9e**) as a colorless oil (0.036 g, 34%), ^{1}H NMR (400 MHz, $CDCl_3$): δ_H 3.53 (2H, m, morpholine H), 3.66 (6H, m, morpholine H), 3.80 (3H, s, OCH_3), 3.84 (3H, s, OCH_3), 3.87 (3H, s, OCH_3), 3.97 (3H, s, OCH_3), 5.16 (2H, s, NCH_2), 5.81 (1H, brs, ArOH), 6.68 (1H, d, *J* 8.7 Hz, ArH), 6.81 (3H, m, 3×ArH), 7.17 (1H, s, ArH); ^{13}C NMR (100 MHz, $CDCl_3$): δ_C 42.4, 45.3, 49.6, 56.0, 56.3, 61.2, 61.4, 66.4, 66.9, 97.3, 98.0, 111.6, 115.7, 120.6, 124.3, 124.9, 127.6, 136.2, 139.1, 140.0, 145.4, 146.3, 149.8, 166.5. HRMS (ESI): calc. for $C_{24}H_{28}N_2NaO_7Se$ $[M+Na]^+$ 559.0954, found 559.0995.

2-Methoxy-5-(5,6,7-trimethoxy-2-methyl-1*H*-indol-3-ylthio)phenol (**10f**, Table 4, entry 5)

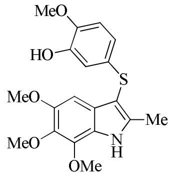

Using method A, compound **10f** was obtained from 3-(3-(allyloxy)-4-methoxyphenylthio)-5,6,7-trimethoxy-2-methyl-1*H*-indole (**9f**) as a pale yellow oil (0.062 g, 83%), ^{1}H NMR (400 MHz, $CDCl_3$): δ_H 2.45 (3H, s, CH_3), 3.79 (3H, s, OCH_3), 3.83 (3H, s, OCH_3), 3.89 (3H, s, OCH_3), 4.06 (3H, s, OCH_3), 5.67 (1H, brs, OH), 6.62 (1H, s, ArH), 6.63 (1H, dd, *J* 7.5, 2.1 Hz, ArH), 6.69 (1H, d, *J* 7.5 Hz, ArH), 6.76 (1H, s, ArH), 8.48 (1H, brs, ArH); ^{13}C NMR (100 MHz, $CDCl_3$): δ_C 12.1, 56.1, 56.4, 61.2, 61.4, 95.8, 100.2, 111.3, 112.7, 117.7, 123.2, 126.4, 131.5, 137.8, 138.4, 140.7, 144.5, 146.0, 149.8. HRMS (ESI): calc. for $C_{19}H_{22}NO_5S$ $[M+H]^+$ 376.1213, found 376.1233. IR (KBr, *v* cm^{-1}): 3333, 2930, 2836, 1723, 1631, 1583, 1499, 1463, 1113, 900, 858.

Ethyl 2-(3-(3-hydroxy-4-methoxyphenylthio)-5,6,7-trimethoxy-1*H*-indol-1-yl)acetate (**10g**, Table 4, entry 6)

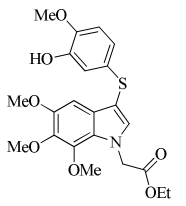

Using method A, compound **10g** was obtained from ethyl 2-(3-(3-(allyloxy)-4-methoxyphenylthio)-5,6,7-trimethoxy-1*H*-indol-1-yl)acetate (**9g**) as a colorless oil which will slowly solidified upon standing (0.046 g, 51%), ^{1}H NMR (400 MHz, $CDCl_3$): δ_H 1.27 (3H, t, *J* 7.1 Hz, CH_3), 3.81 (3H, s, OCH_3), 3.83 (3H, s, OCH_3), 3.87 (3H, s, OCH_3), 3.97 (3H, s, OCH_3), 4.24 (2H, q, *J* 7.1 Hz, OCH_2), 5.02 (2H, s, NCH_2), 5.63 (1H, brs, OH), 6.70 (3H, m, 3×ArH), 6.68 (1H, s, ArH), 7.16 (1H, s, ArH); ^{13}C NMR (100 MHz, $CDCl_3$): δ_C 14.2, 50.2, 56.1, 56.2, 61.2, 61.3, 61.5, 96.1, 102.7, 111.3, 113.1, 118.0, 124.3, 126.7, 131.1, 135.5, 139.1, 140.3, 144.7, 146.0, 150.0, 169.0. HRMS (ESI): calc. for $C_{22}H_{25}NNaO_7S$ $[M+Na]^+$ 470.1244, found 470.1246.

2-(3-(3-Hydroxy-4-methoxyphenylthio)-5,6,7-trimethoxy-1*H*-indol-1-yl)-1-morpholinoethanone (**10h**, Table 4, entry 7)

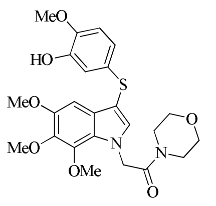

Using method A, compound **10h** was obtained from 2-(3-(3-(allyloxy)-4-methoxyphenylthio)-5,6,7-trimethoxy-1*H*-indol-1-yl)-1-morpholinoethanone (**9h**) as a colorless oil which will slowly solidified upon standing (0.09 g, 93%), ^{1}H NMR (400 MHz, $CDCl_3$): δ_H 3.55 (2H, m, morpholine H), 3.68 (6H, m, morpholine H), 3.81 (3H, s, OCH_3), 3.82 (3H, s, OCH_3), 3.87 (3H, s, OCH_3), 3.97 (3H, s, OCH_3), 5.16 (2H, s, NCH_2), 5.75 (1H, brs, ArOH), 6.70 (3H, m, 3×ArH), 6.79 (1H, s, ArH), 7.18 (1H, s, ArH); ^{13}C NMR (100 MHz, $CDCl_3$): δ_C 42.4, 45.3, 49.7, 56.1, 56.3, 61.2, 61.4, 66.5, 66.9, 96.4, 111.3, 113.0, 117.9, 124.4, 126.8, 128.5, 128.6, 131.2, 135.7, 139.2, 140.1, 146.0, 149.9, 166.4. MS (ESI, pos) 489.2 $[M+H]^+$, 511.2 $[M+Na]^+$, 527.1 $[M+K]^+$, MS (ESI, neg) 487.0 $[M-H]^-$. HRMS (ESI): calc. for $C_{24}H_{28}N_2NaO_7S$ $[M+Na]^+$ 511.1509, found 511.1508; calc. for $C_{24}H_{29}N_2O_7S$ $[M+H]^+$ 489.1690, found 489.1687.

^{1}H NMR and ^{13}C NMR spectra of the starting materials and products

Methyl 3,4,5-trimethoxybenzoate

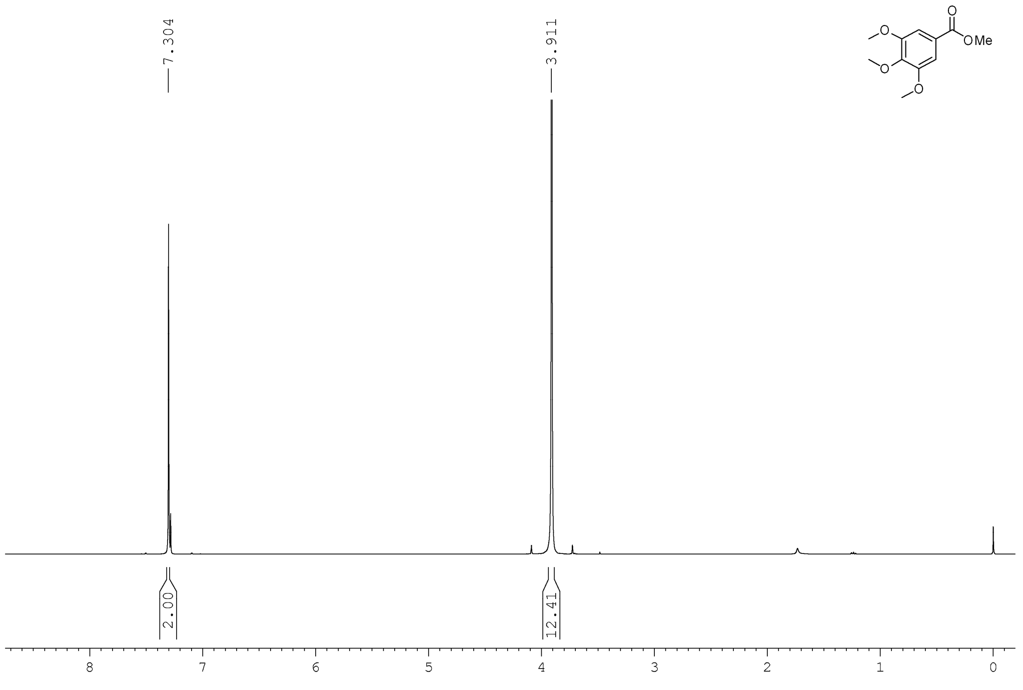

Methyl 3,4,5-trimethoxy-2-nitrobenzoate

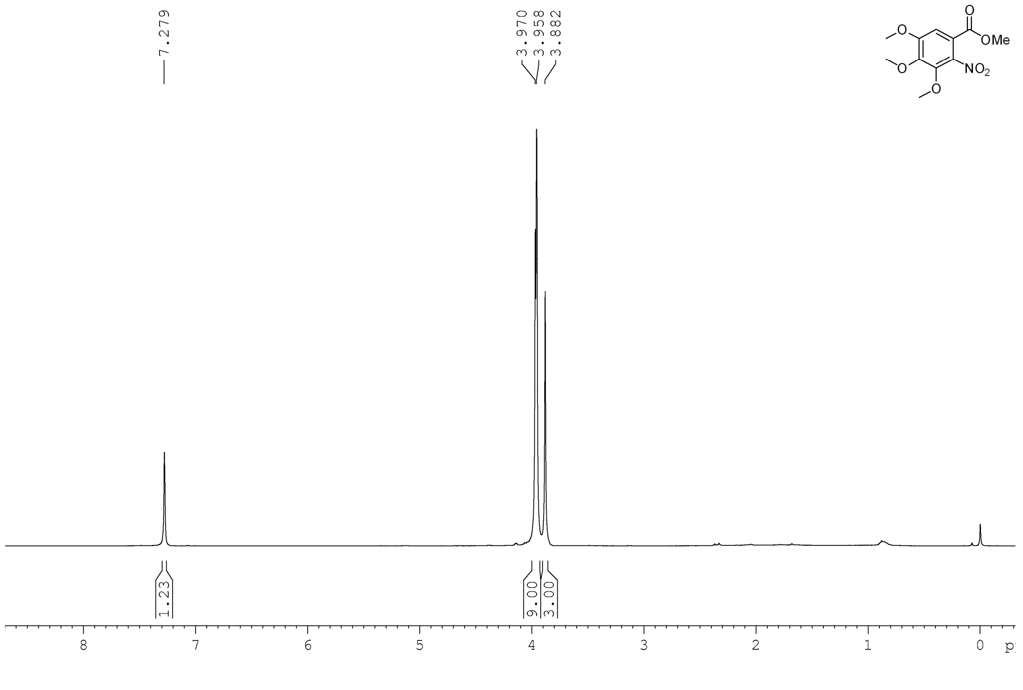

(3,4,5-Trimethoxy-2-nitrophenyl)methanol

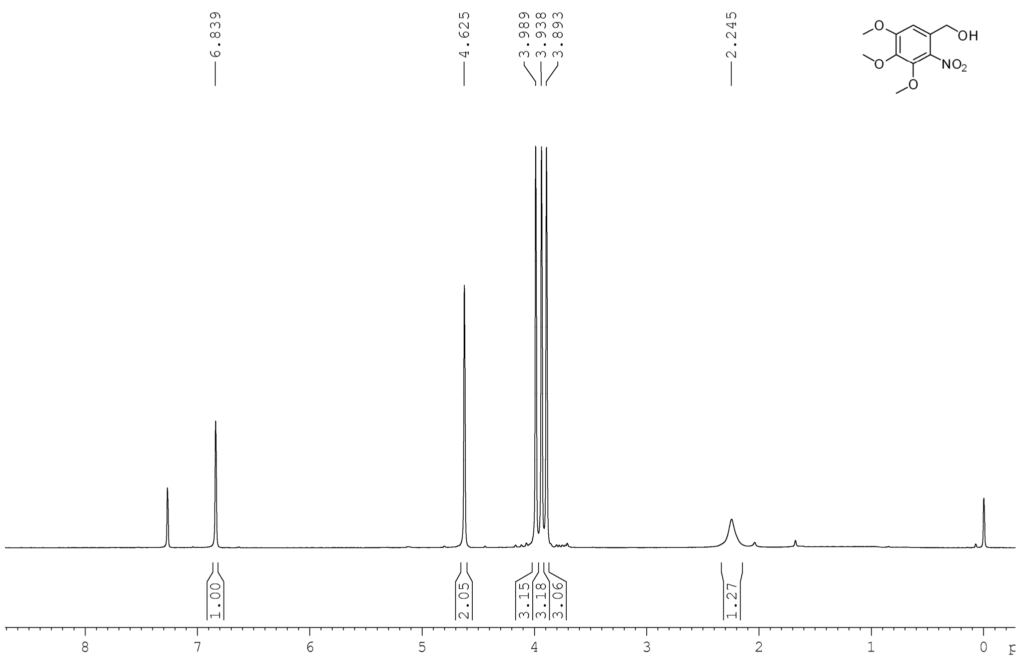

3,4,5-Trimethoxy-2-nitrobenzaldehyde

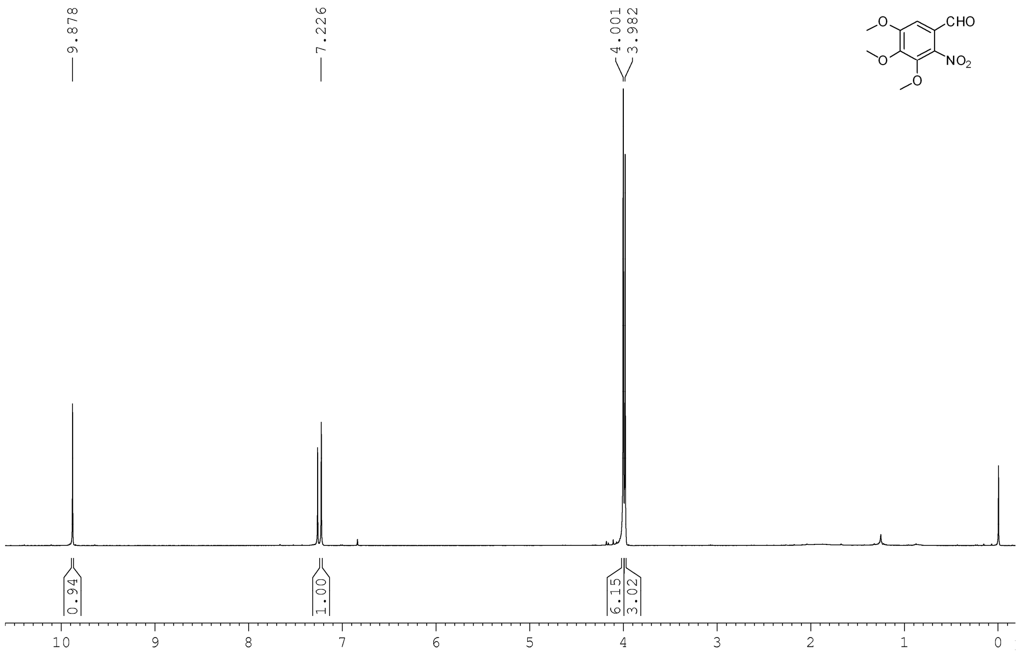

1,2,3-Trimethoxy-4-nitro-5-(2-nitroprop-1-enyl)benzene

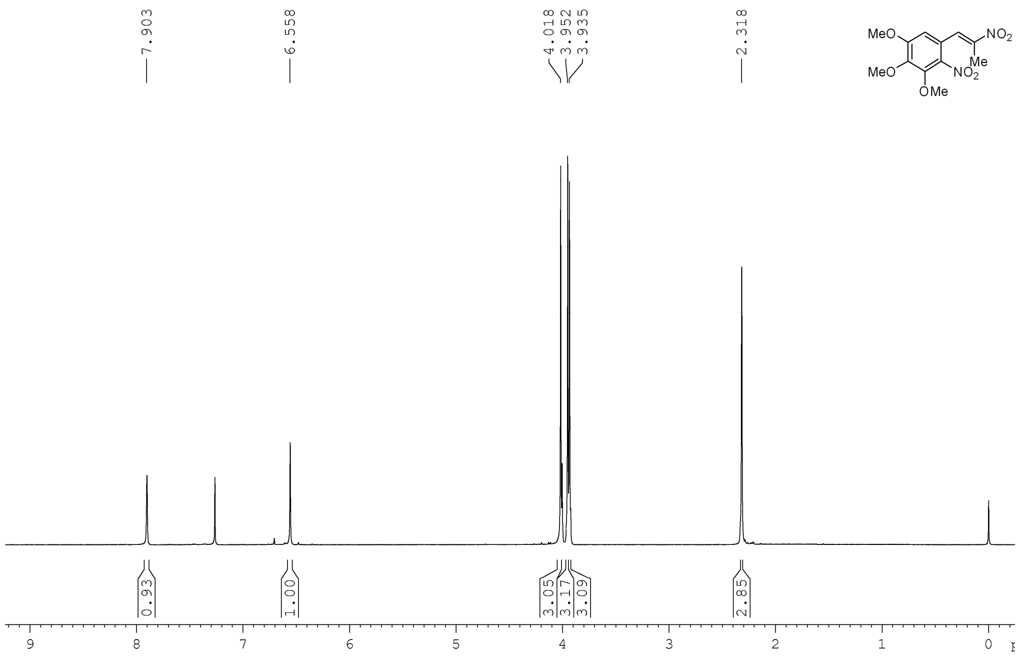

5,6,7-Trimethoxy-2-methyl-1H-indole (**7a**)

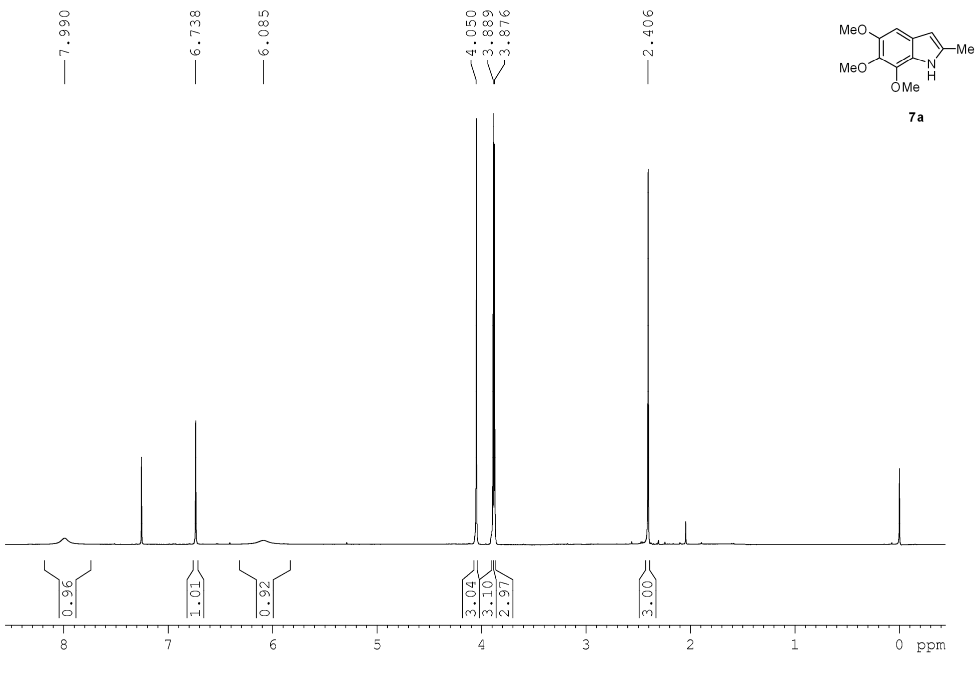

(Z)-1,2,3-Trimethoxy-4-nitro-5-(2-nitrovinyl)benzene

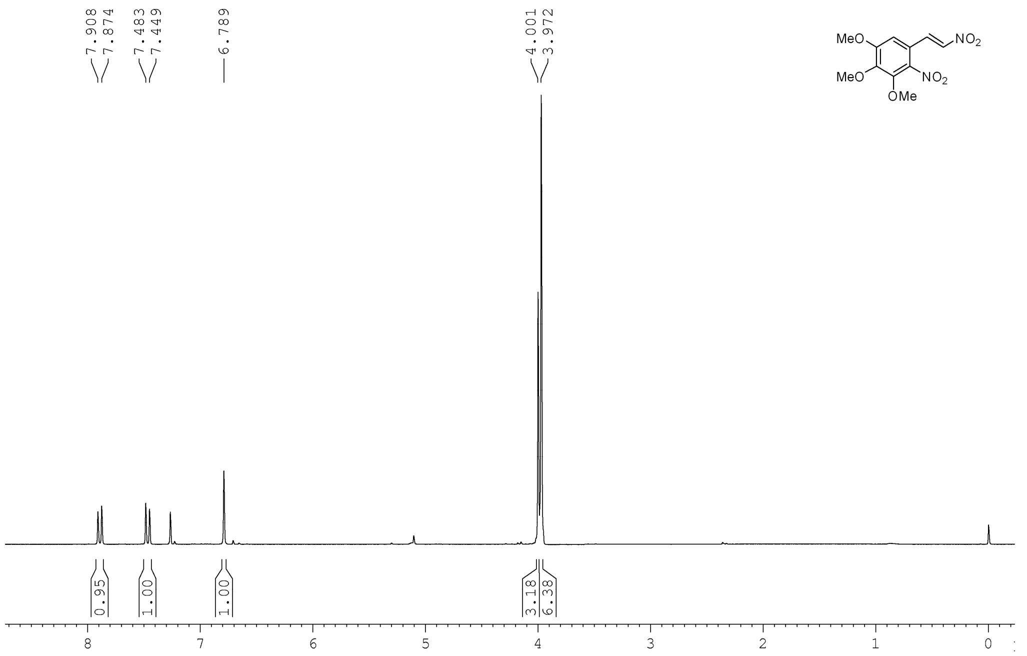

5,6,7-Trimethoxy-1*H*-indole (**7b**)

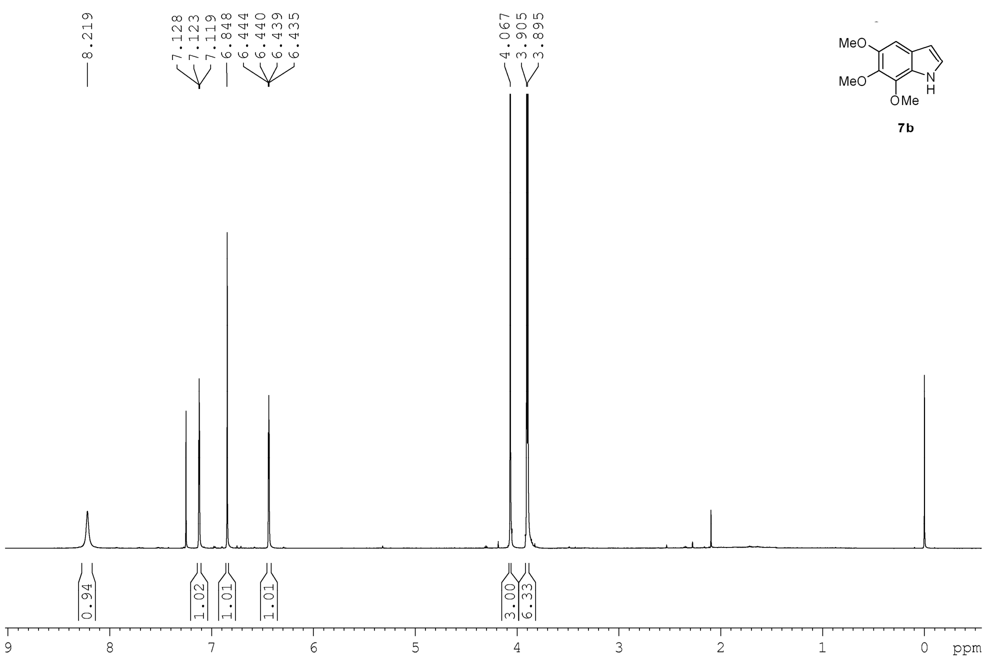

1-(4-Chlorobenzyl)-5,6,7-trimethoxy-1*H*-indole (**7c**)

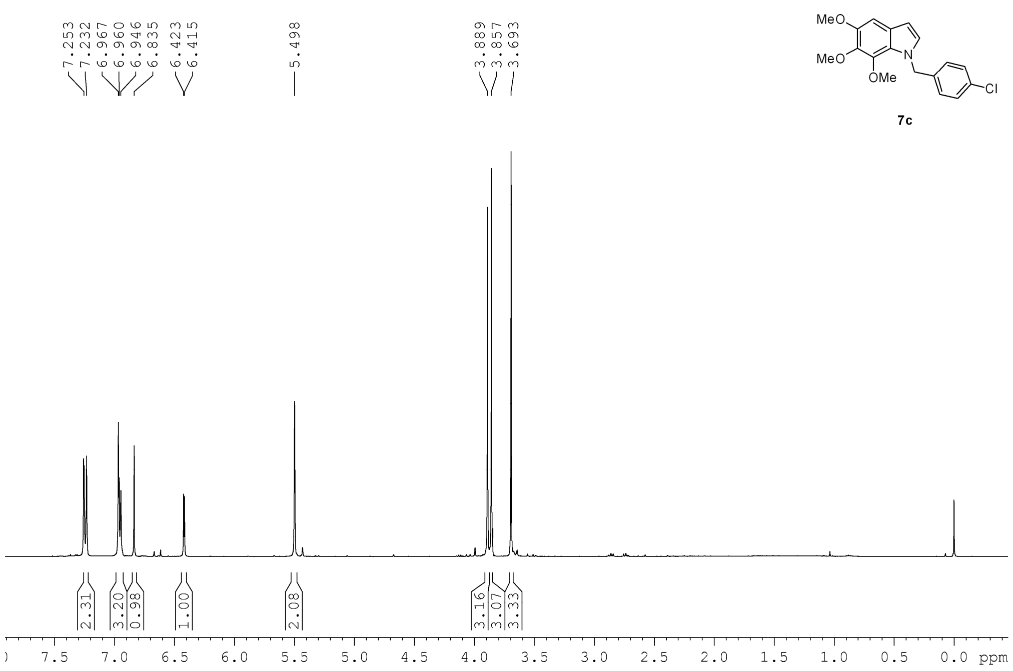

Ethyl 2-(5,6,7-trimethoxy-1*H*-indol-1-yl)acetate (**7d**)

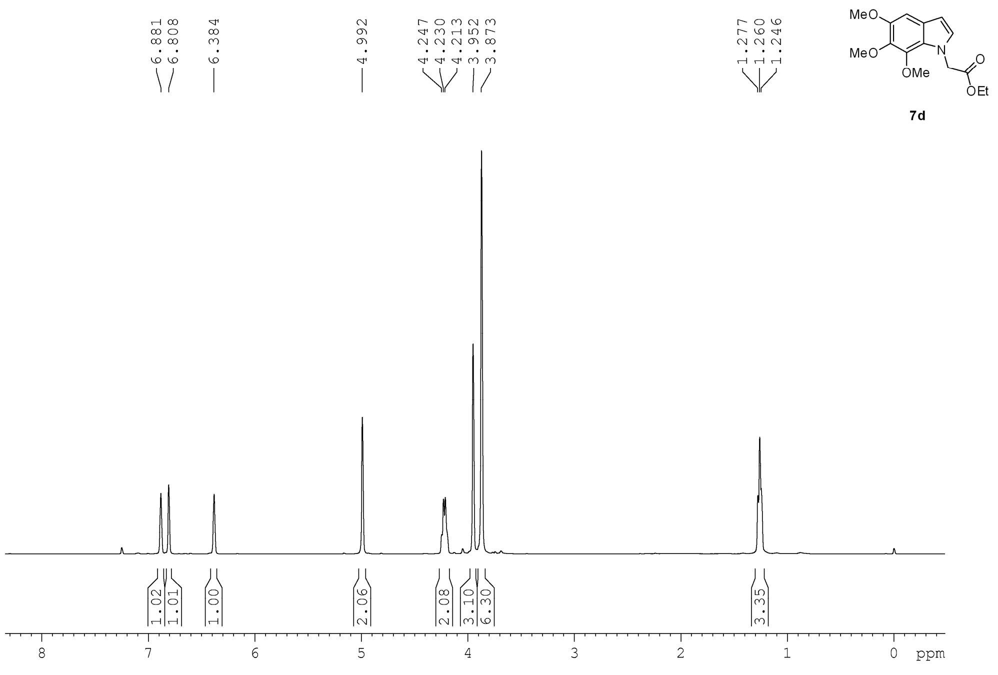

1-Morpholino-2-(5,6,7-trimethoxy-1*H*-indol-1-yl)ethanone (**7e**)

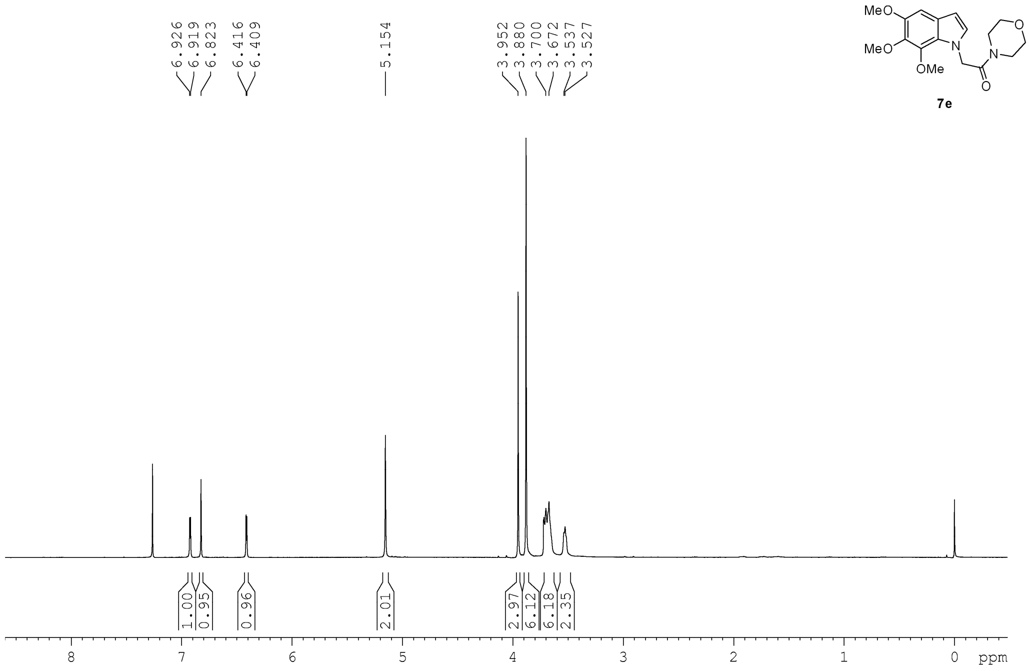

1,2-bis(3-(allyloxy)-4-methoxyphenyl)diselenide (**8a**)

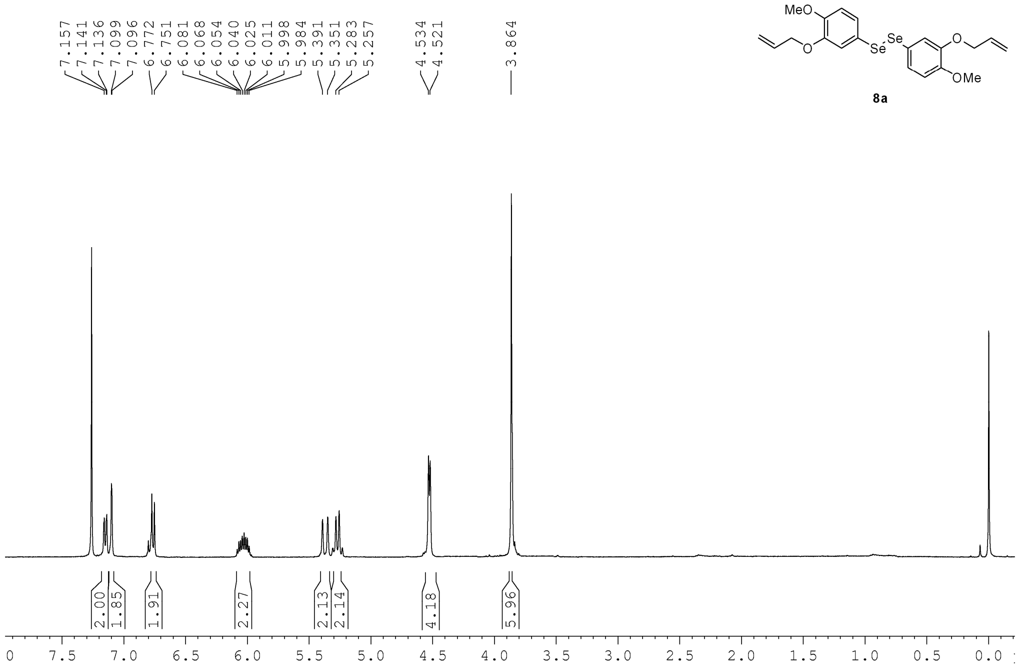

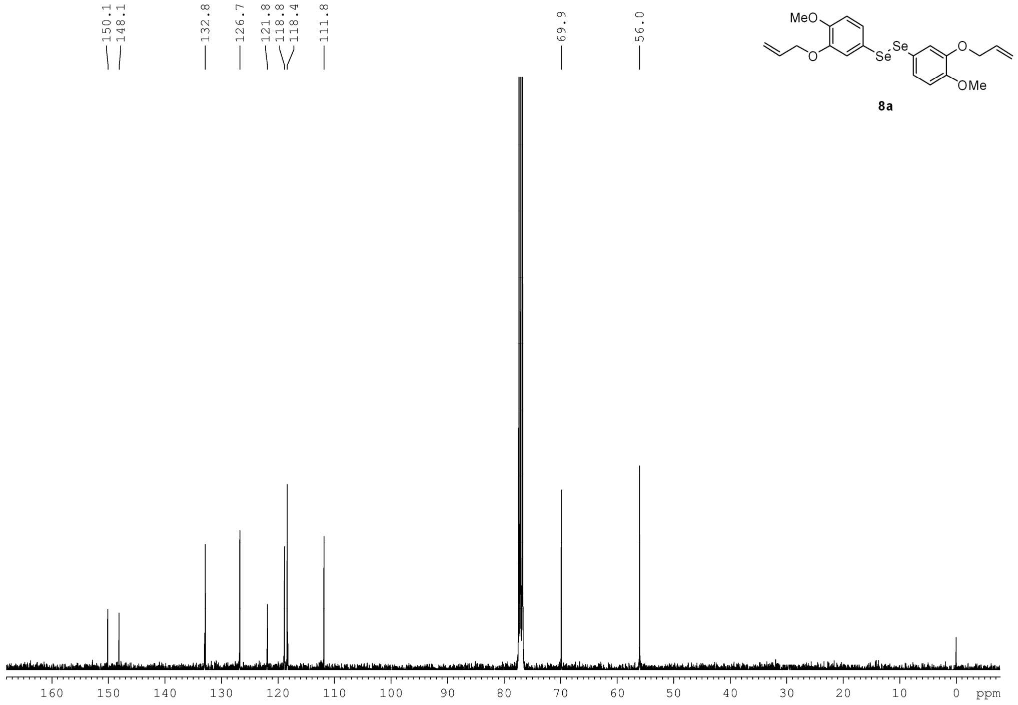

1,2-Bis(3-(allyloxy)-4-methoxyphenyl) disulphide (**8b**)

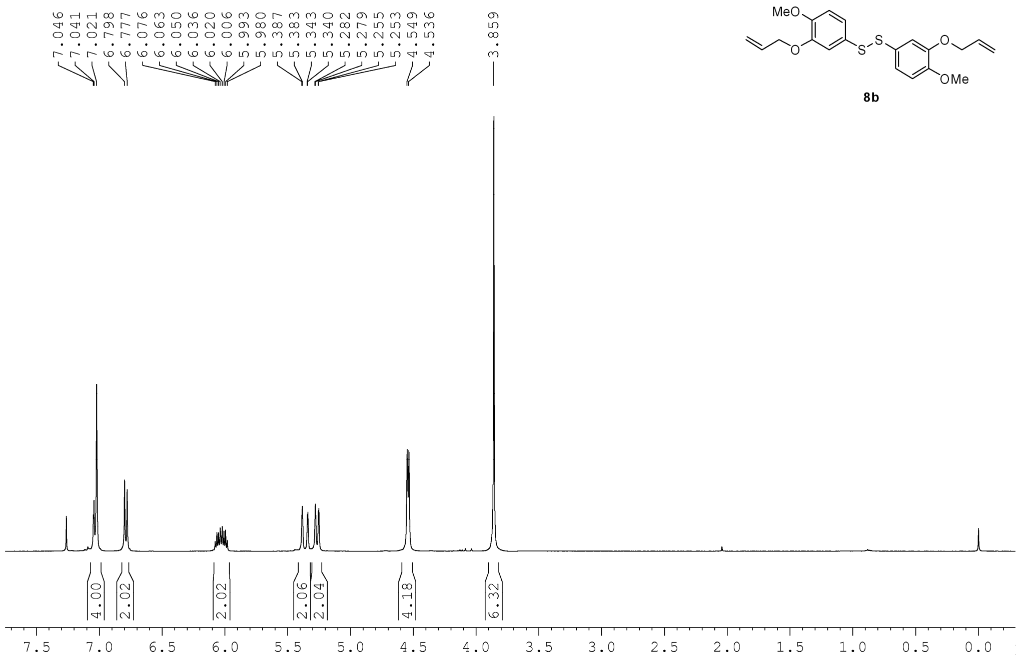

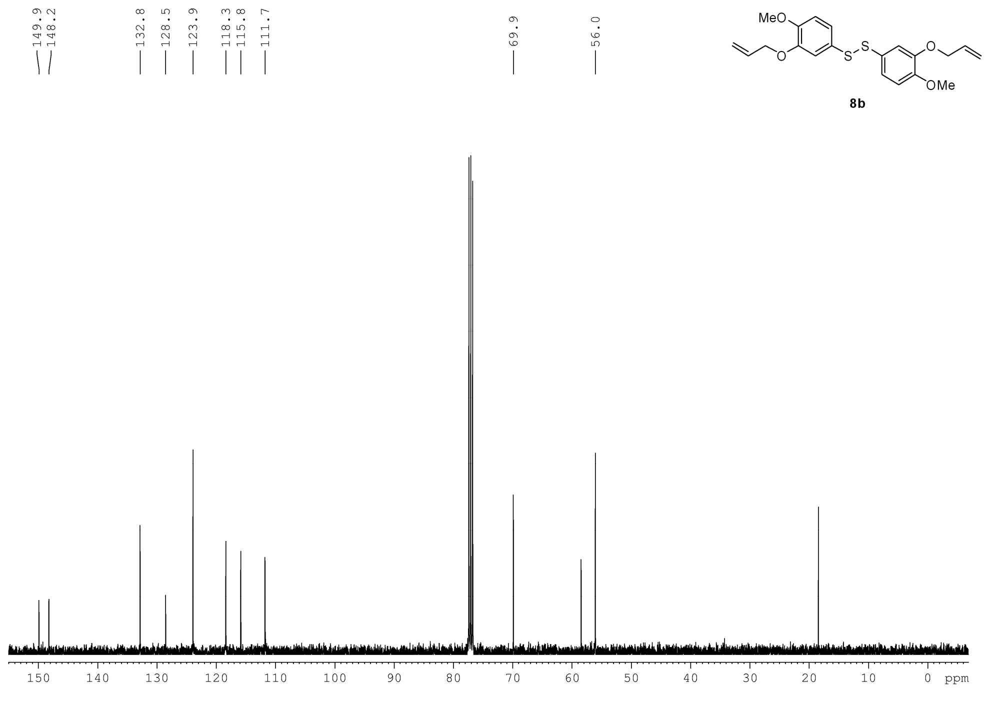

^{1}H NMR and ^{13}C NMR spectra of products 9a-9h

3-((3-(Allyloxy)-4-methoxyphenyl)selenyl)-5,6,7-trimethoxy-2-methyl-1*H*-indole (**9a**)

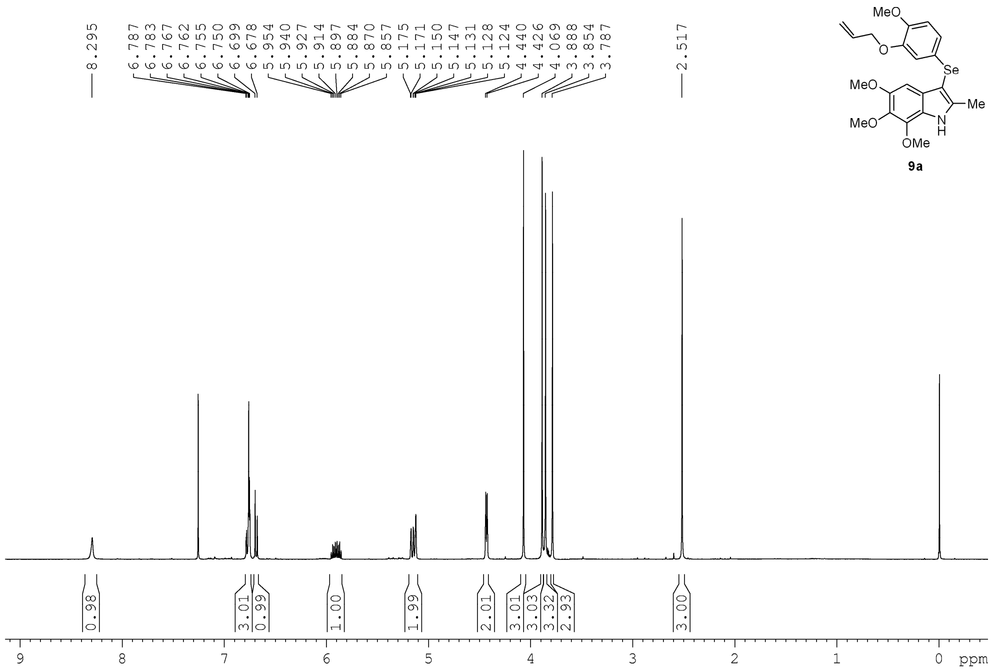

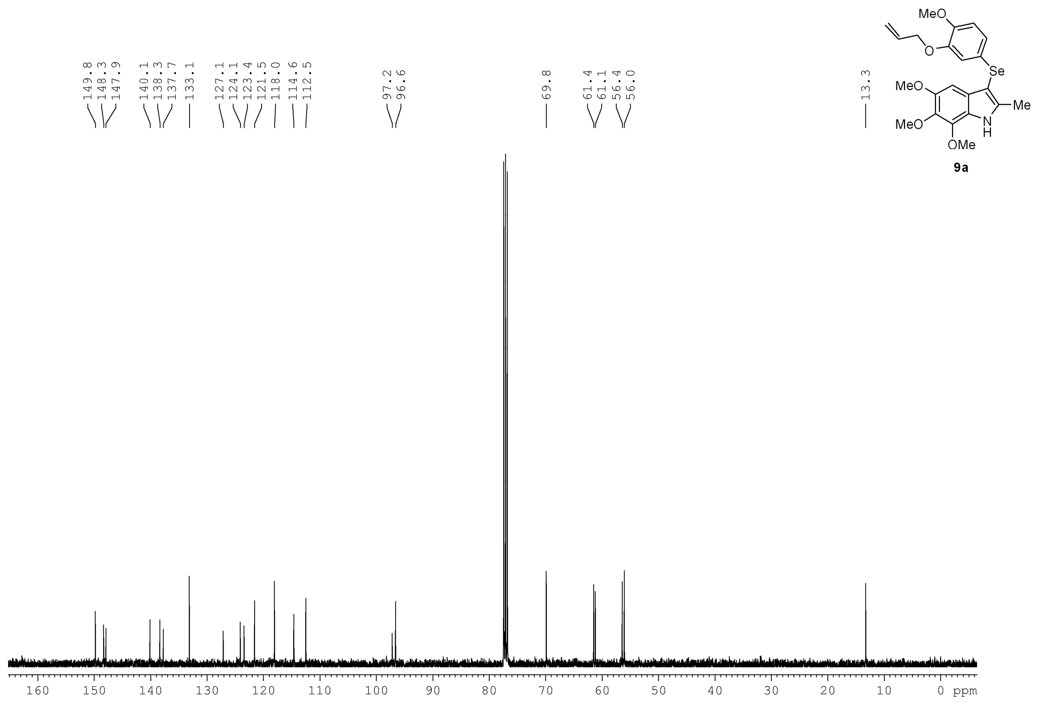

3-(3-(Allyloxy)-4-methoxyphenylselenyl)-5,6,7-trimethoxy-1*H*-indole (**9b**)

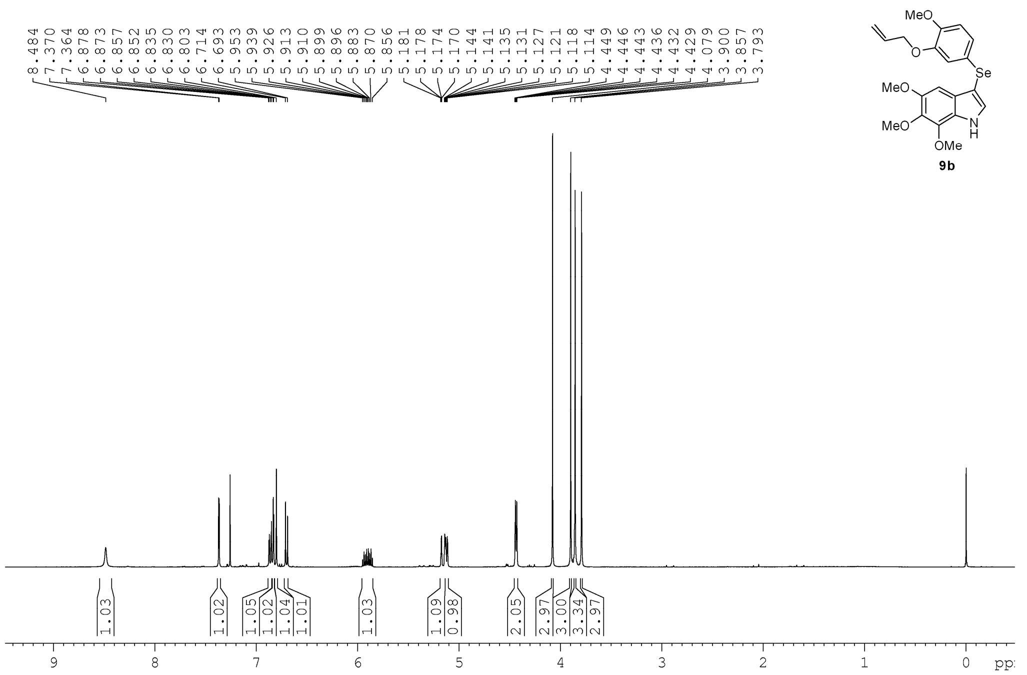

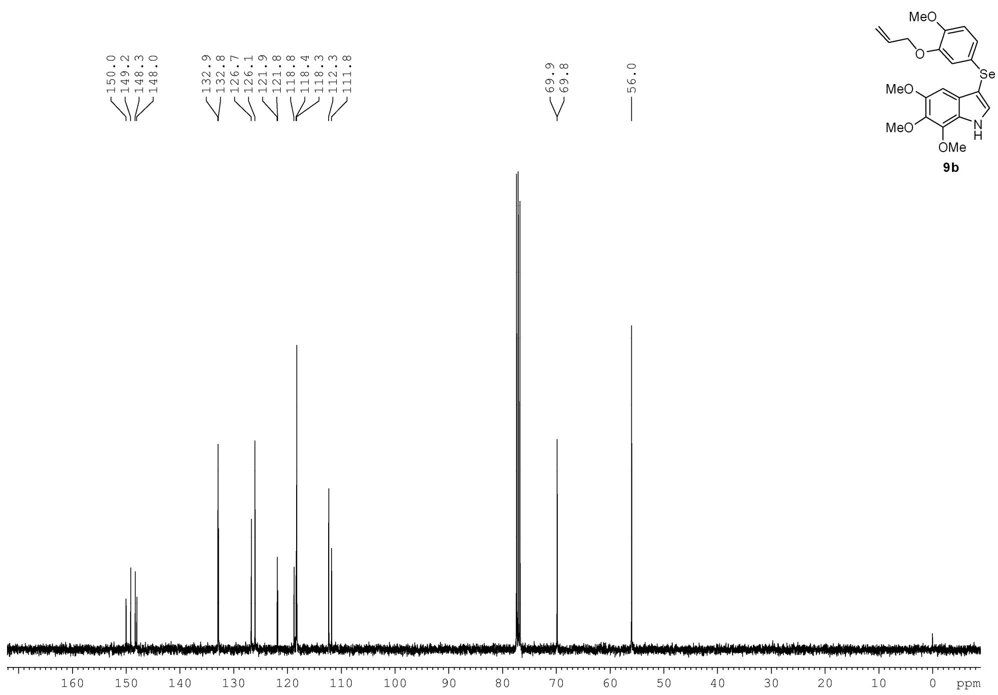

3-(3-(Allyloxy)-4-methoxyphenylselenyl)-1-(4-chlorobenzyl)-5,6,7-trimethoxy-1*H*-indole (**9c**)

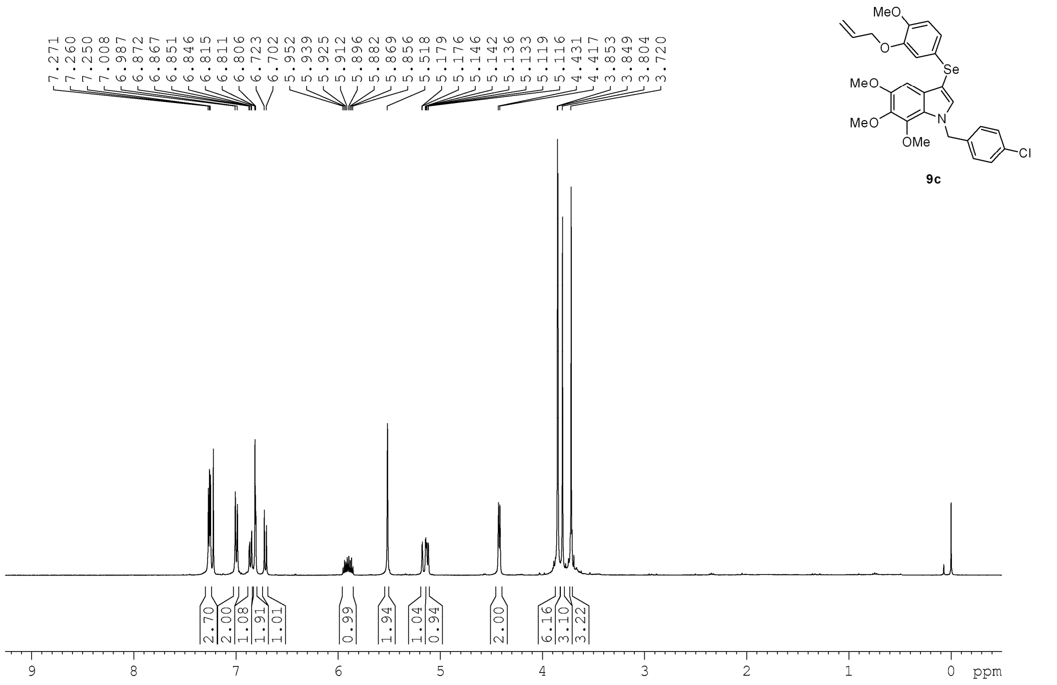

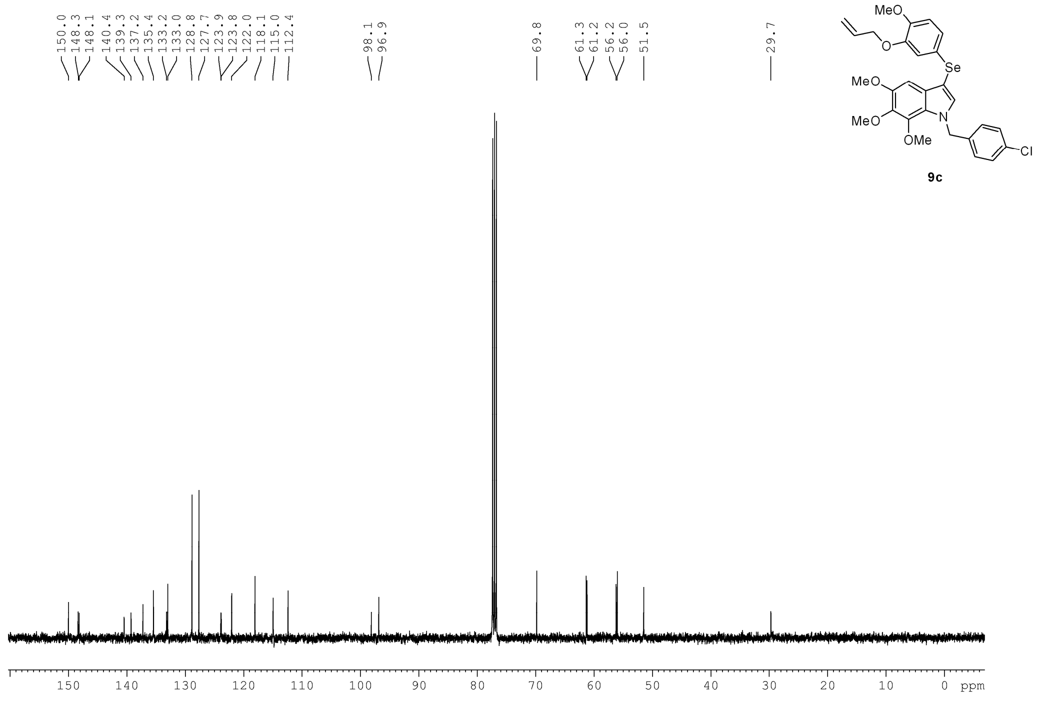

Ethyl 2-(3-(3-(allyloxy)-4-methoxyphenylselenyl)-5,6,7-trimethoxy-1*H*-indol-1-yl)acetate (**9d**)

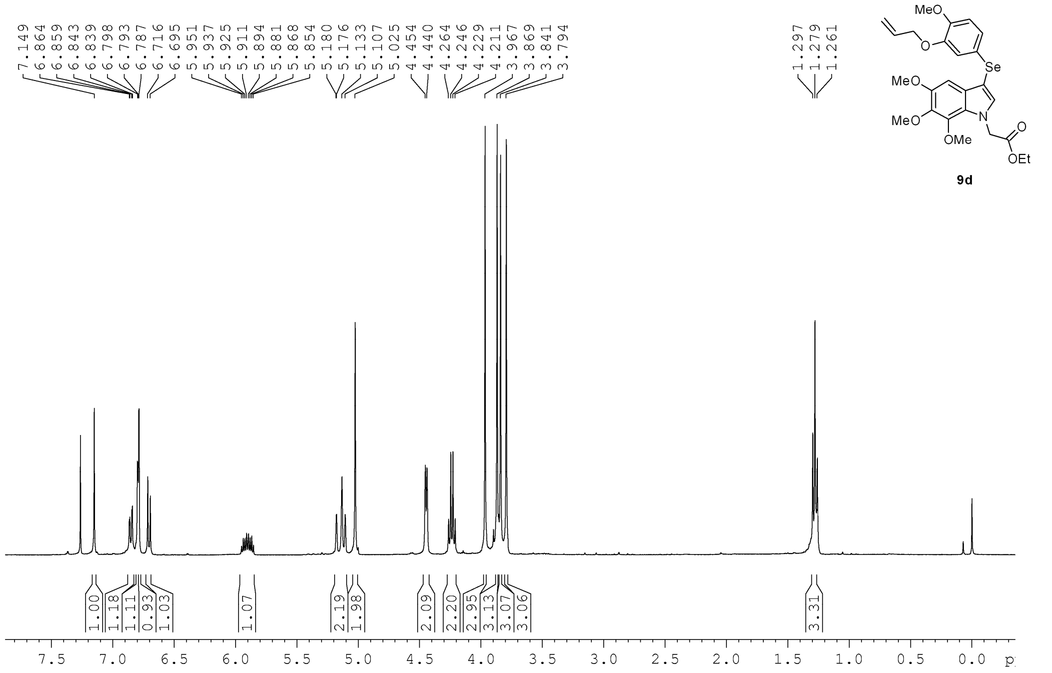

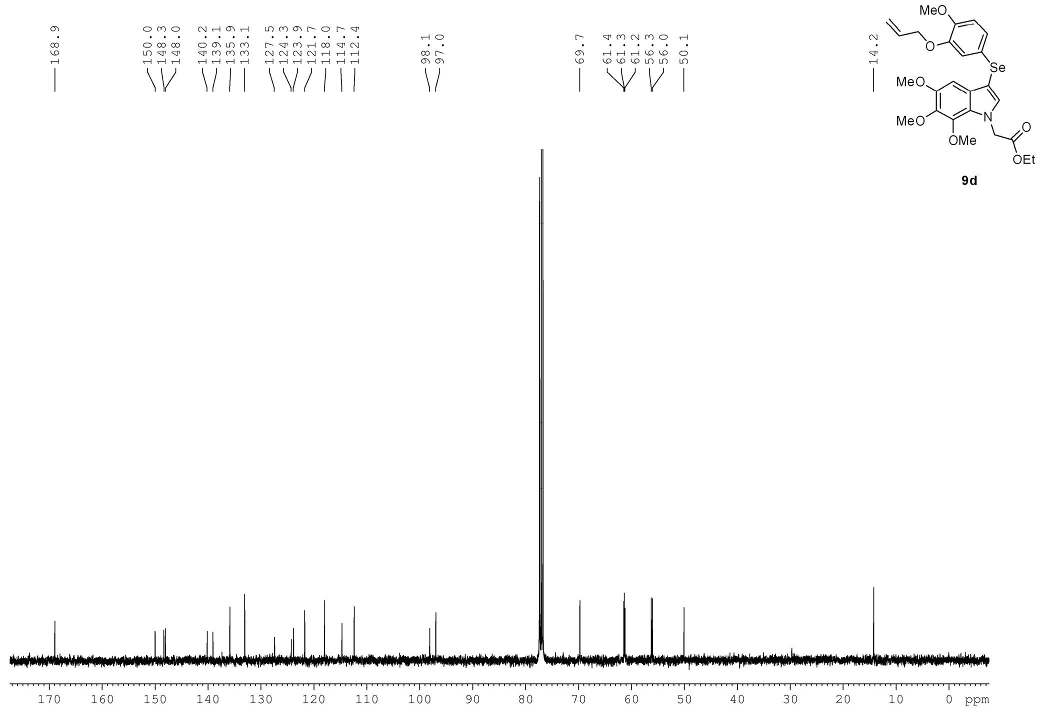

2-(3-(3-(Allyloxy)-4-methoxyphenylselenyl)-5,6,7-trimethoxy-1*H*-indol-1-yl)-1-morpholinoethanone (**9e**)

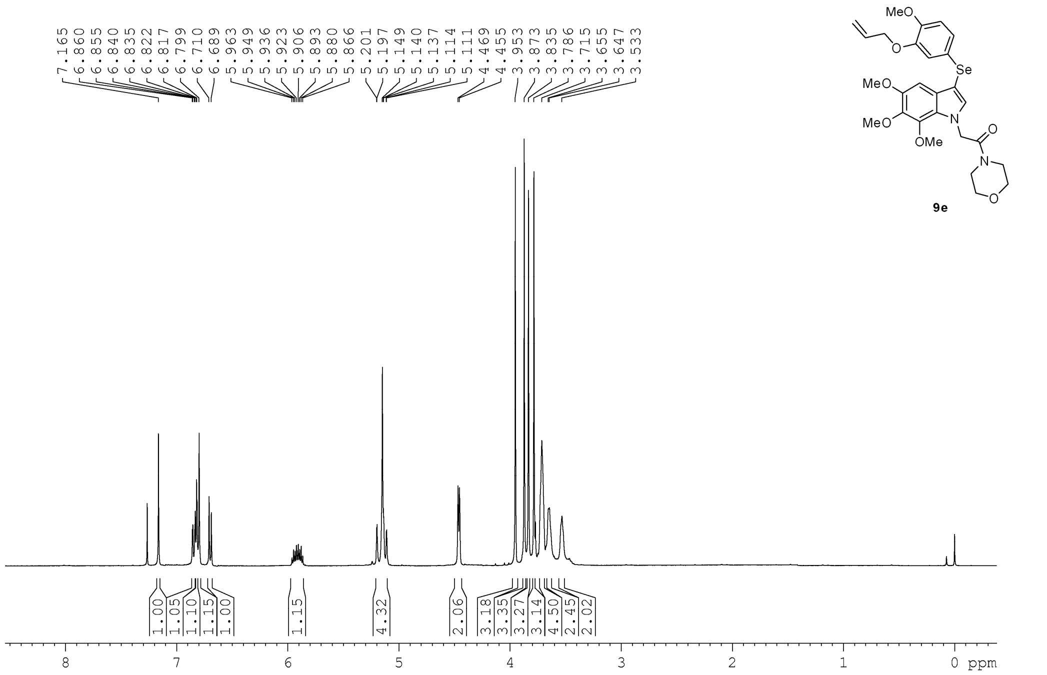

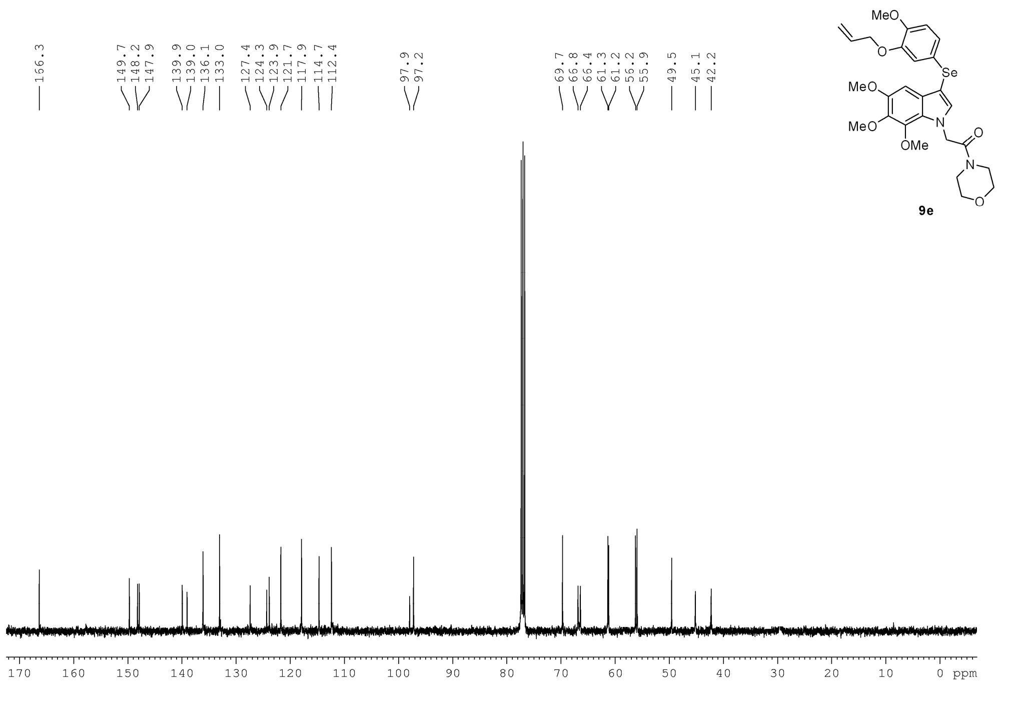

3-(3-(Allyloxy)-4-methoxyphenylthio)-5,6,7-trimethoxy-2-methyl-1*H*-indole (**9f**)

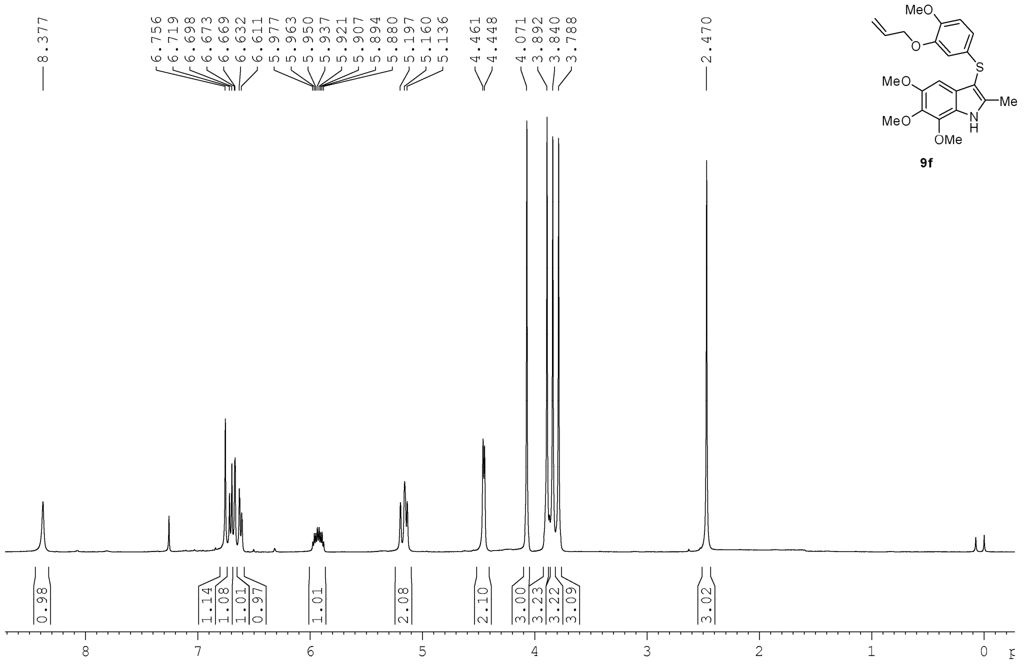

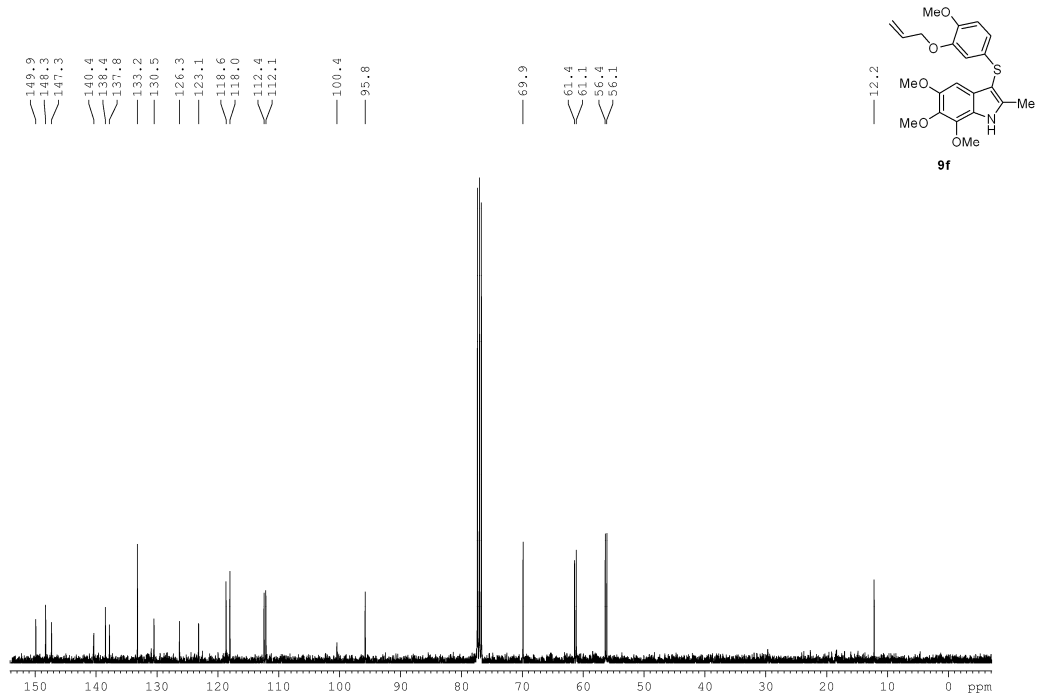

Ethyl 2-(3-(3-(allyloxy)-4-methoxyphenylthio)-5,6,7-trimethoxy-1*H*-indol-1-yl)acetate (**9g**)

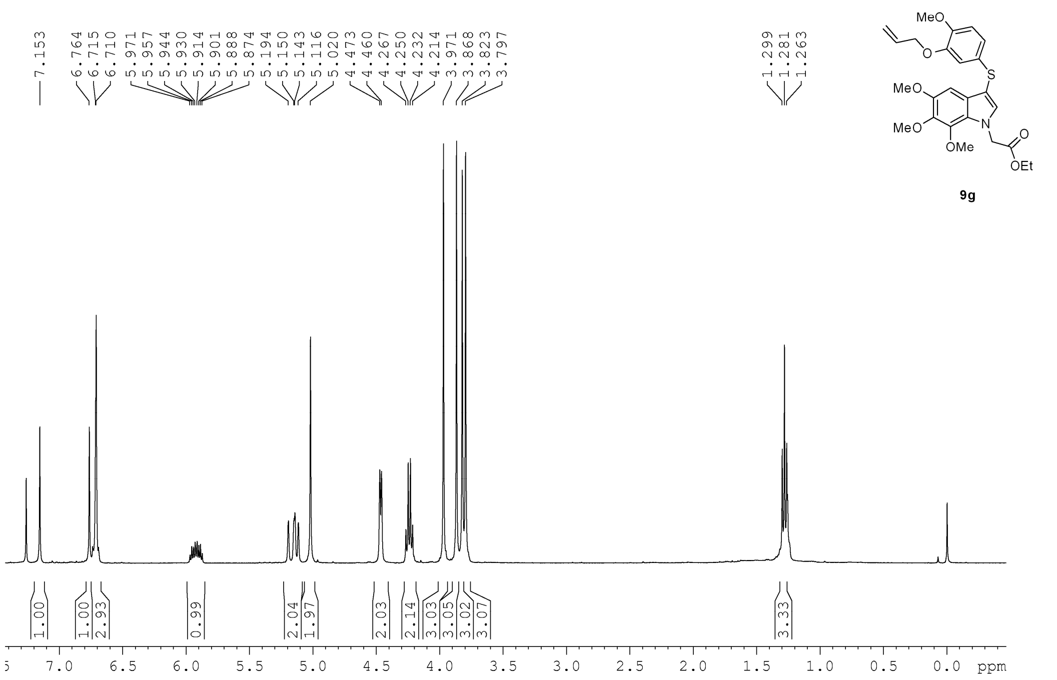

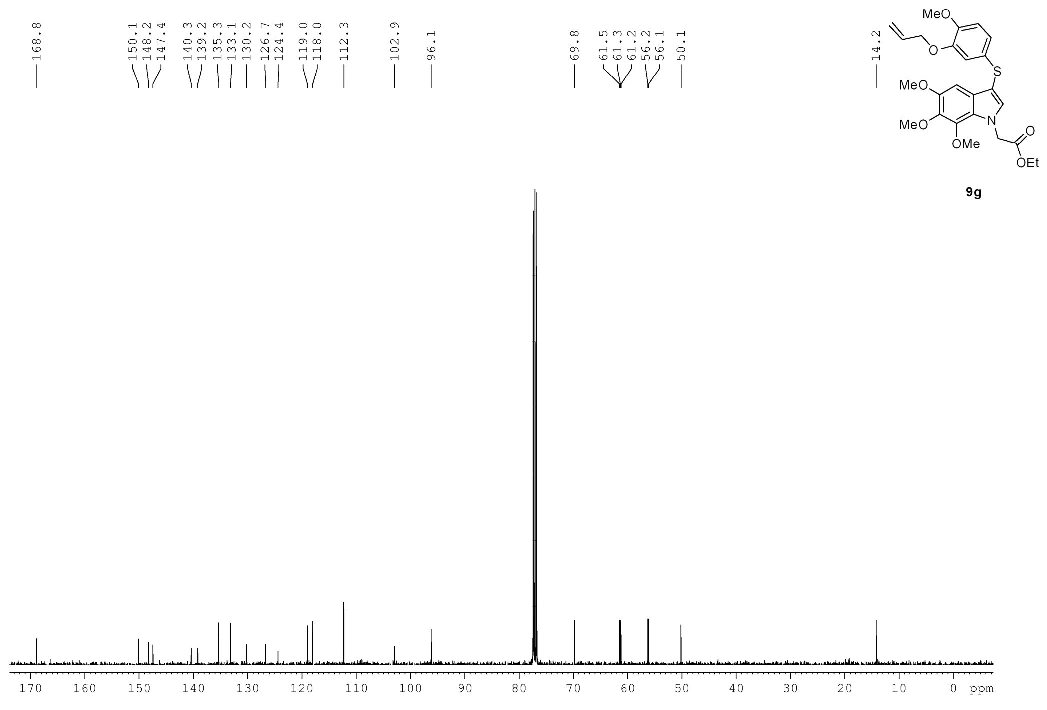

2-(3-(3-(Allyloxy)-4-methoxyphenylthio)-5,6,7-trimethoxy-1*H*-indol-1-yl)-1-morpholinoethanone (**9h**)

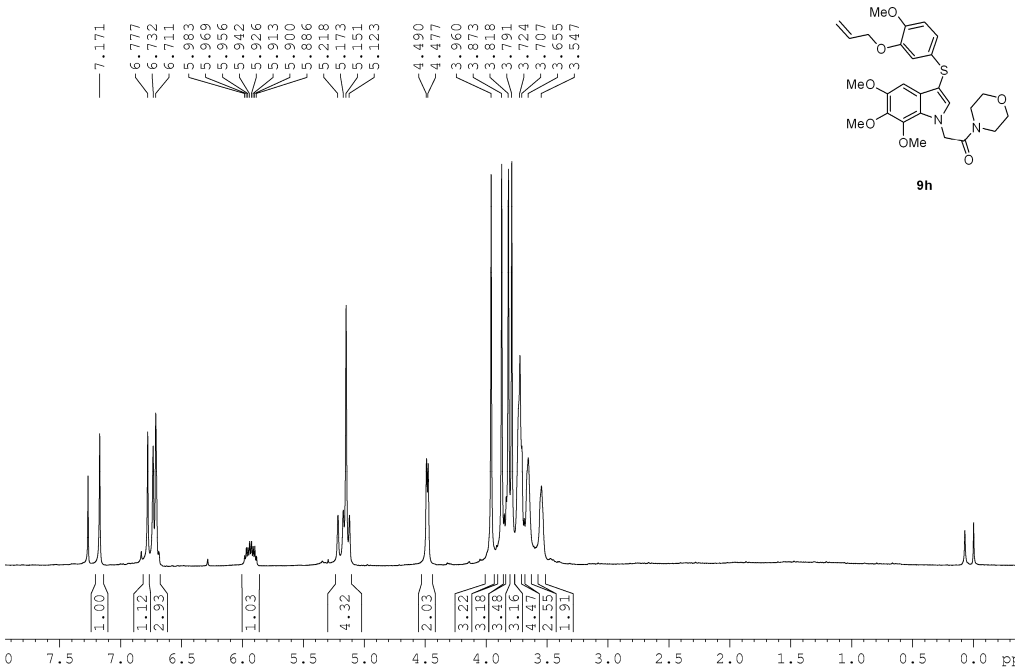

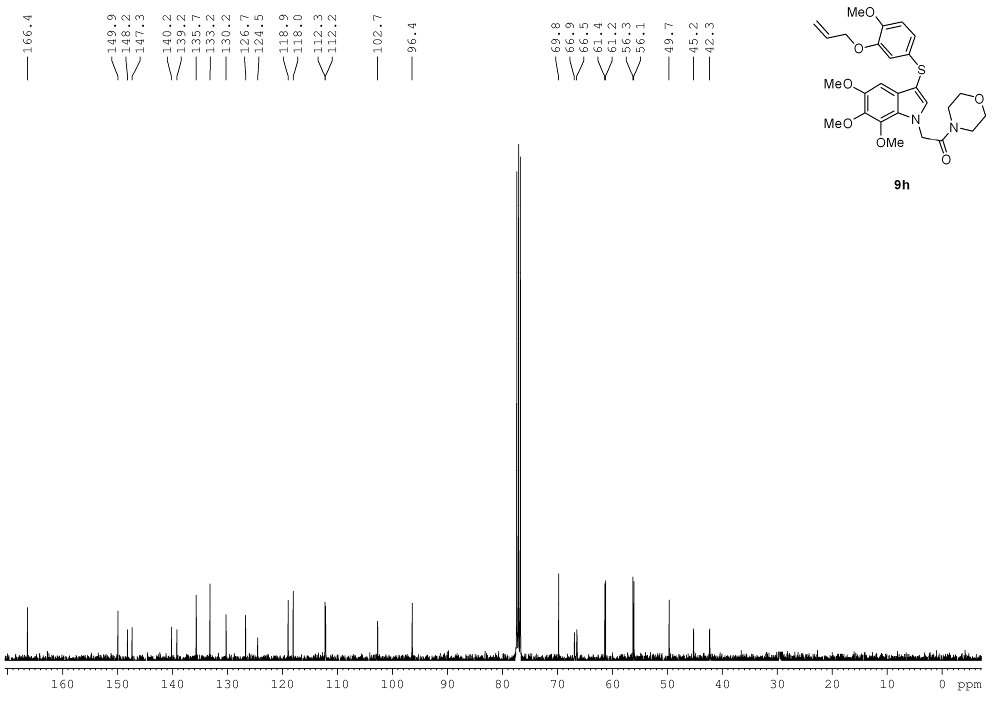

^{1}H NMR and ^{13}C NMR spectra of products 10a-10h

2-Methoxy-5-(5,6,7-trimethoxy-2-methyl-1*H*-indol-3-ylselenyl)phenol (**10a**)

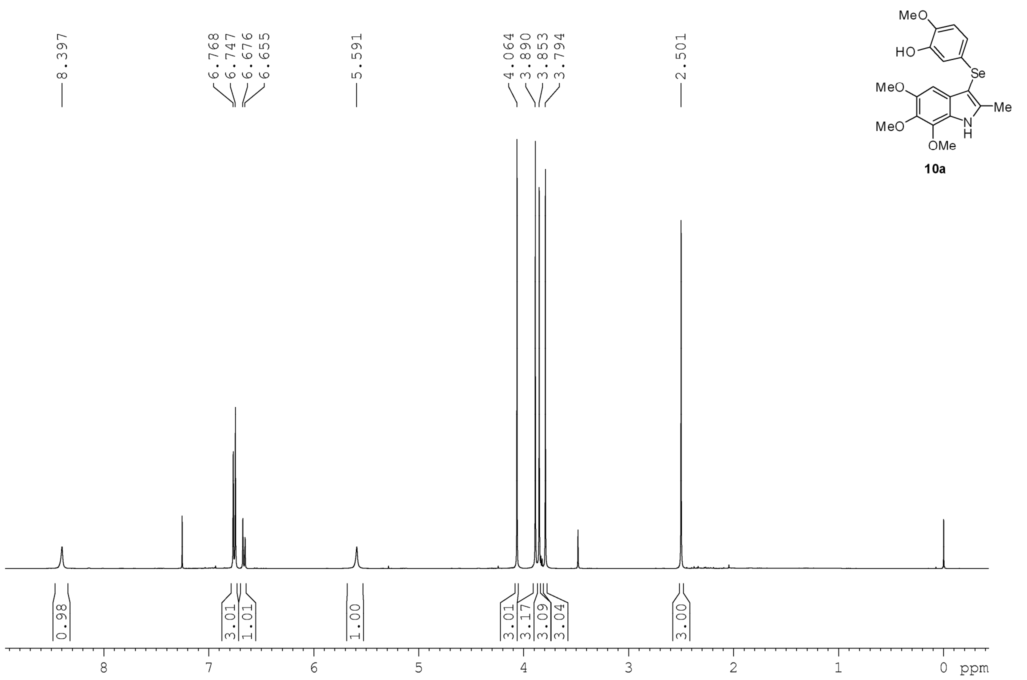

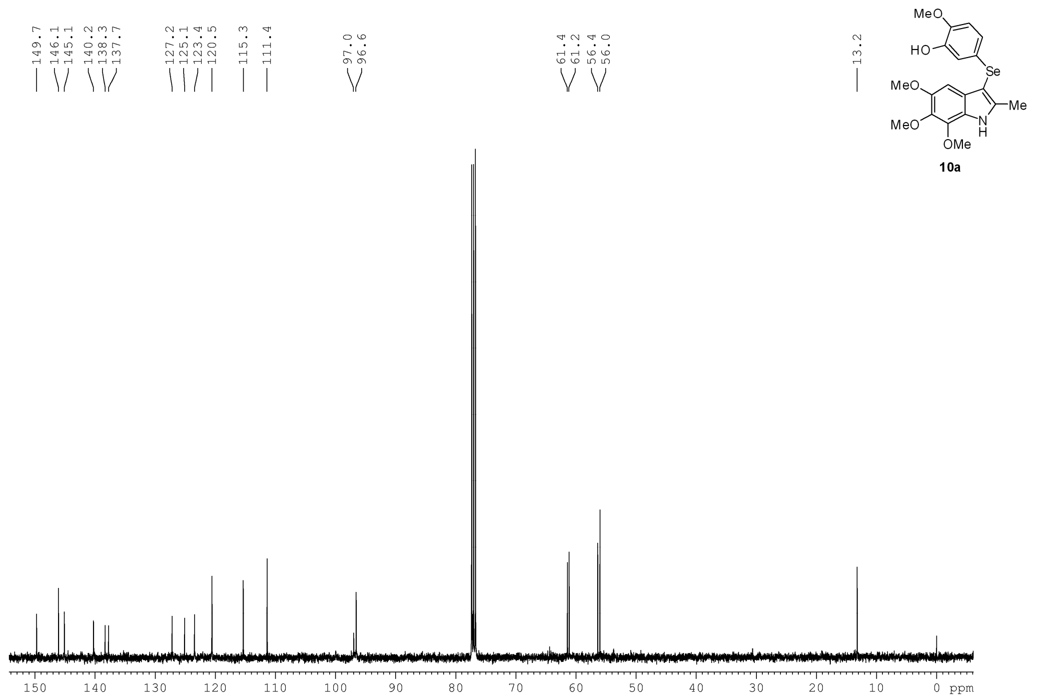

2-Methoxy-5-(5,6,7-trimethoxy-1*H*-indol-3-ylselenyl)phenol (**10b**)

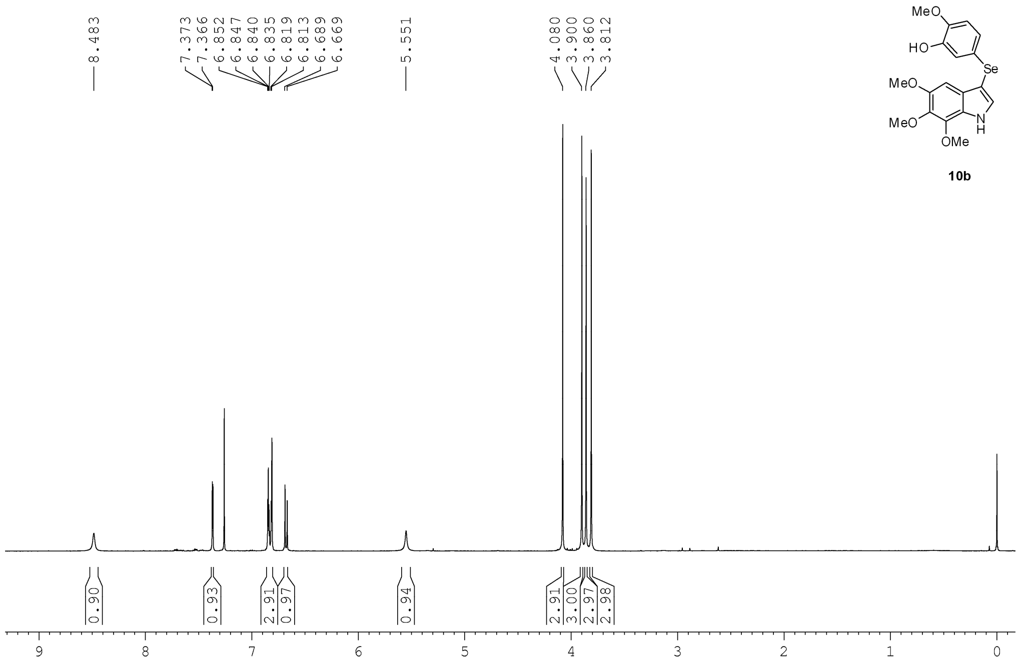

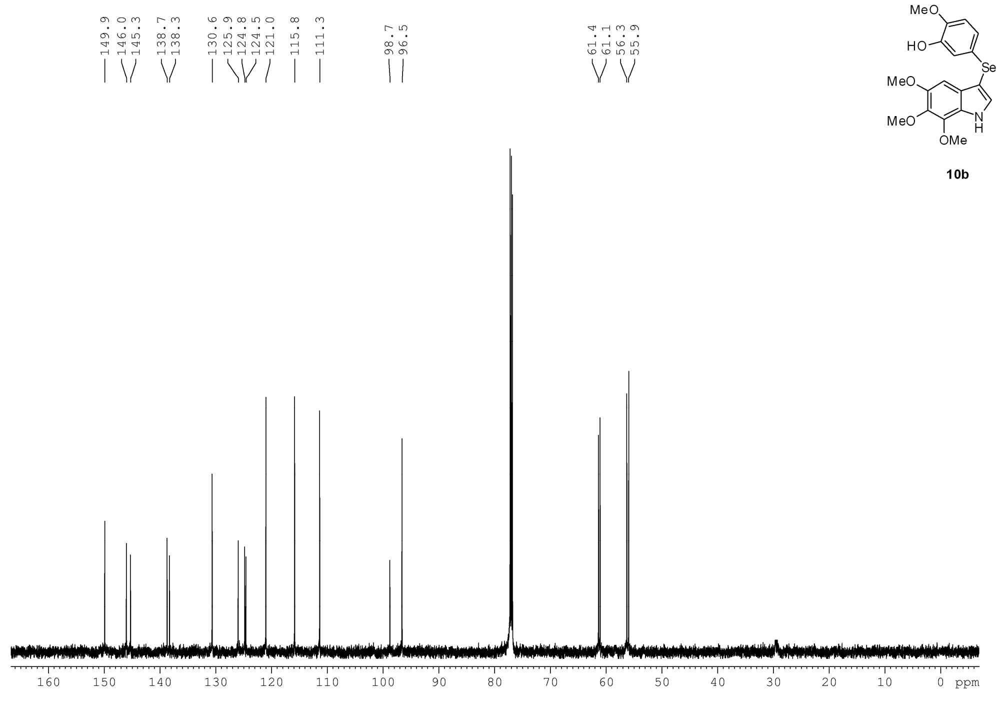

5-(1-(4-Chlorobenzyl)-5,6,7-trimethoxy-1*H*-indol-3-ylselenyl)-2-methoxyphenol (**10c**)

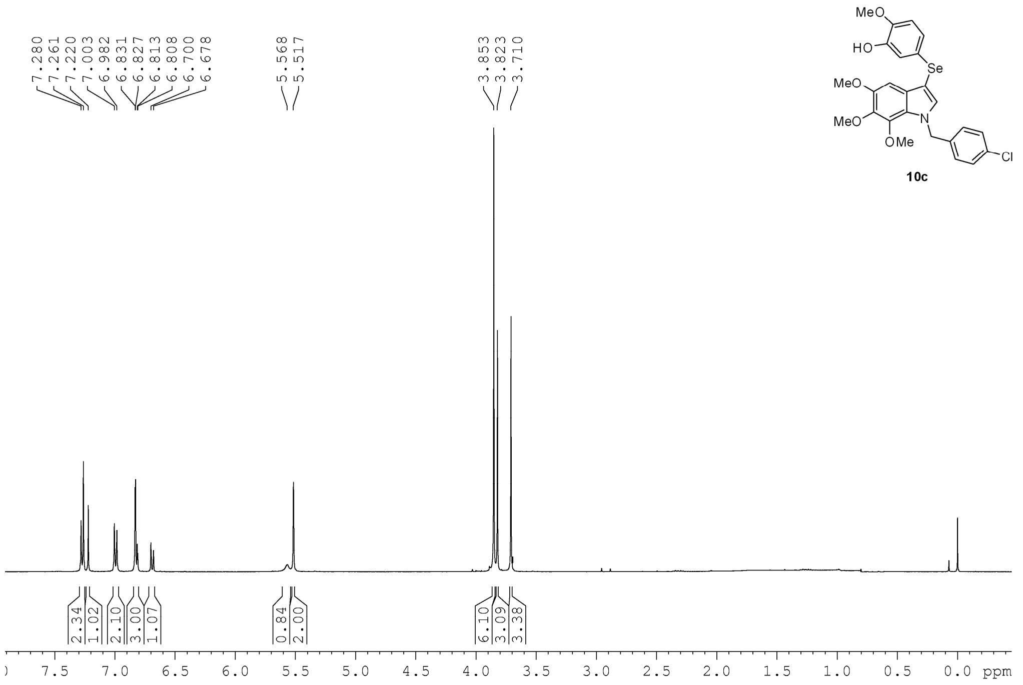

10c

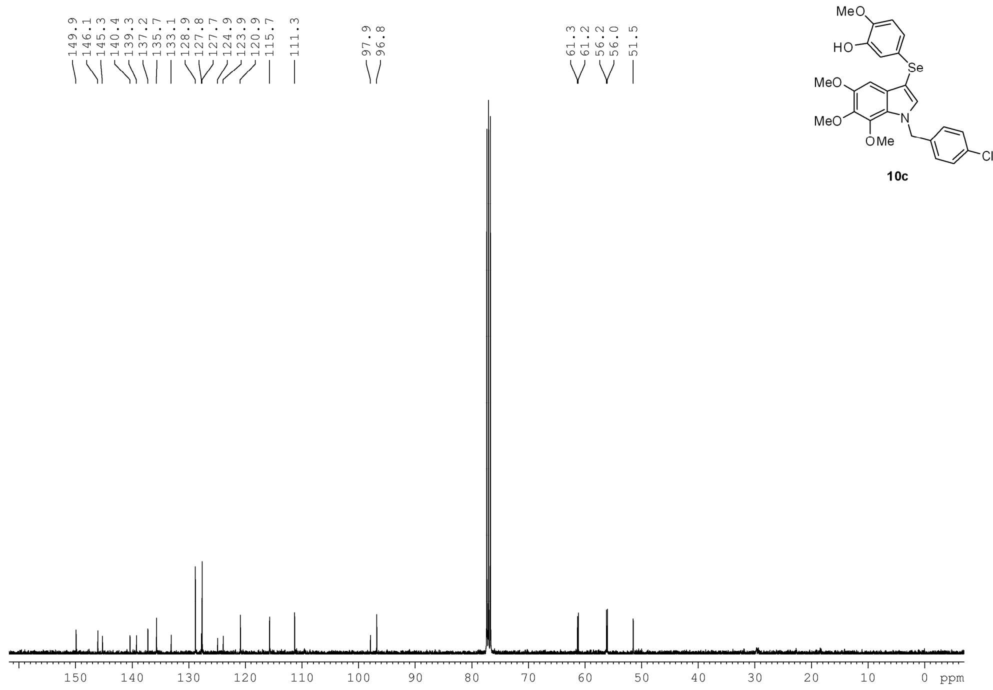

10c

Ethyl 2-(3-(3-hydroxy-4-methoxyphenylselenyl)-5,6,7-trimethoxy-1*H*-indol-1-yl)acetate (**10d**)

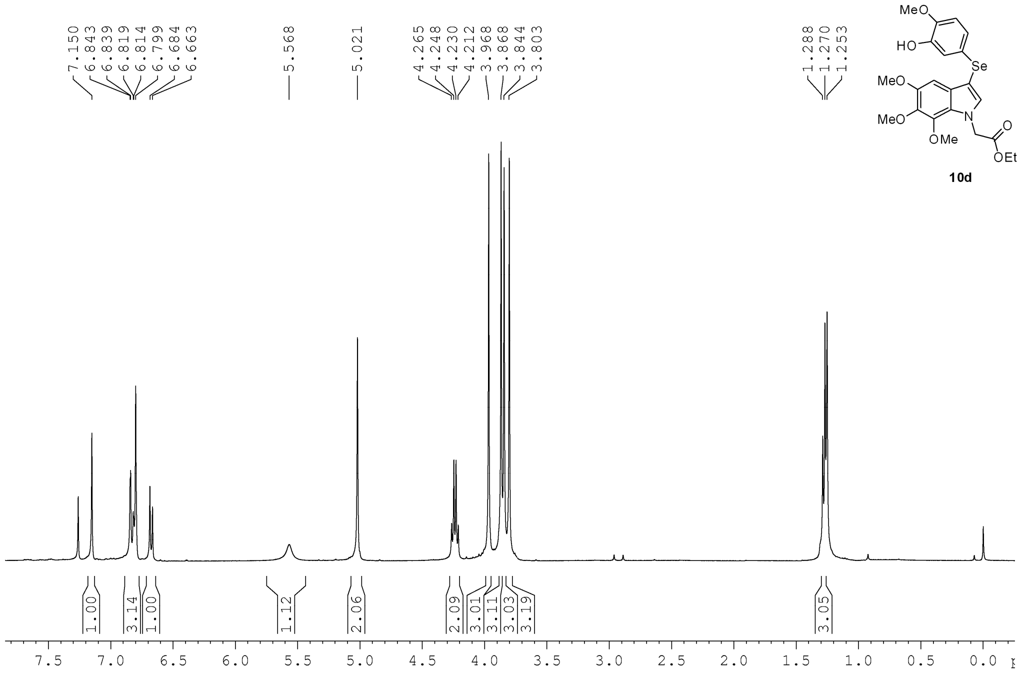

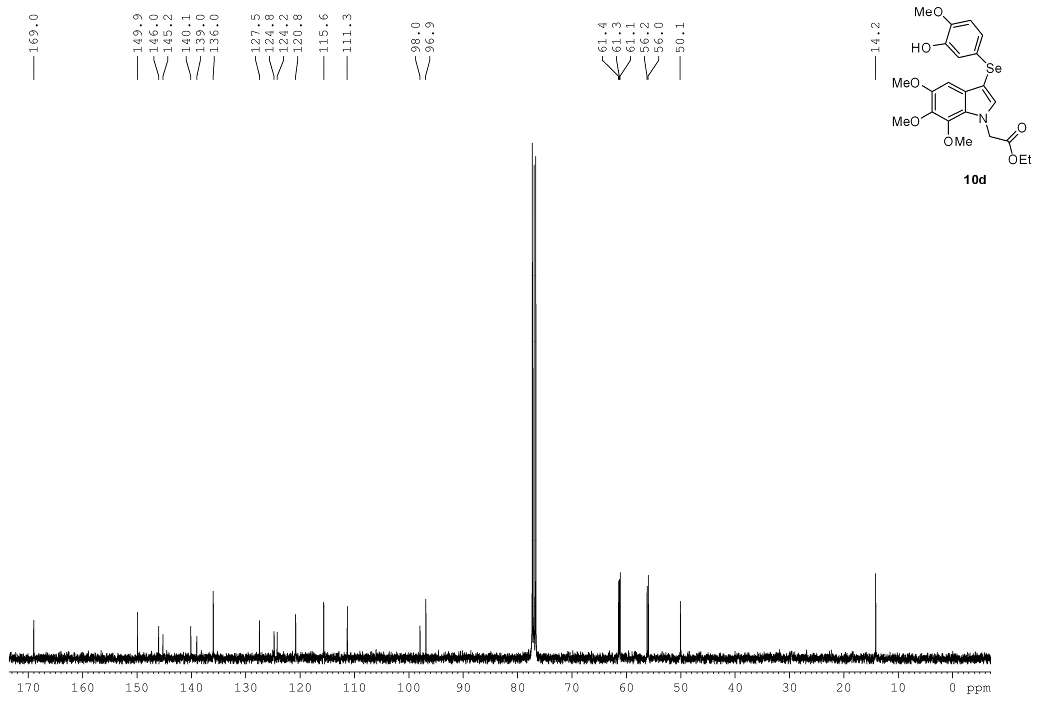

2-(3-(3-Hydroxy-4-methoxyphenylselenyl)-5,6,7-trimethoxy-1*H*-indol-1-yl)-1-morpholinoethanone (**10e**)

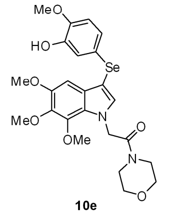

10e

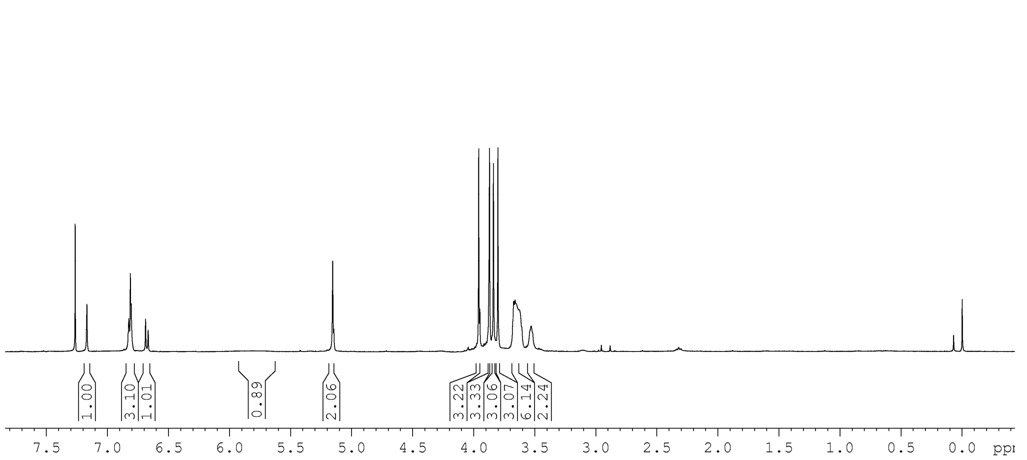

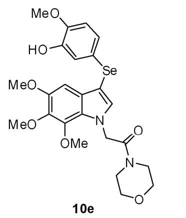

10e

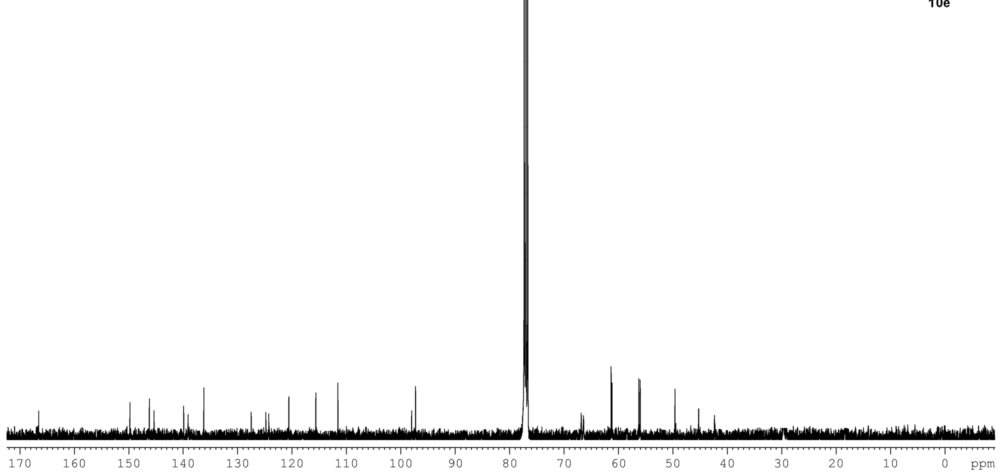

2-Methoxy-5-(5,6,7-trimethoxy-2-methyl-1*H*-indol-3-ylthio)phenol (**10f**)

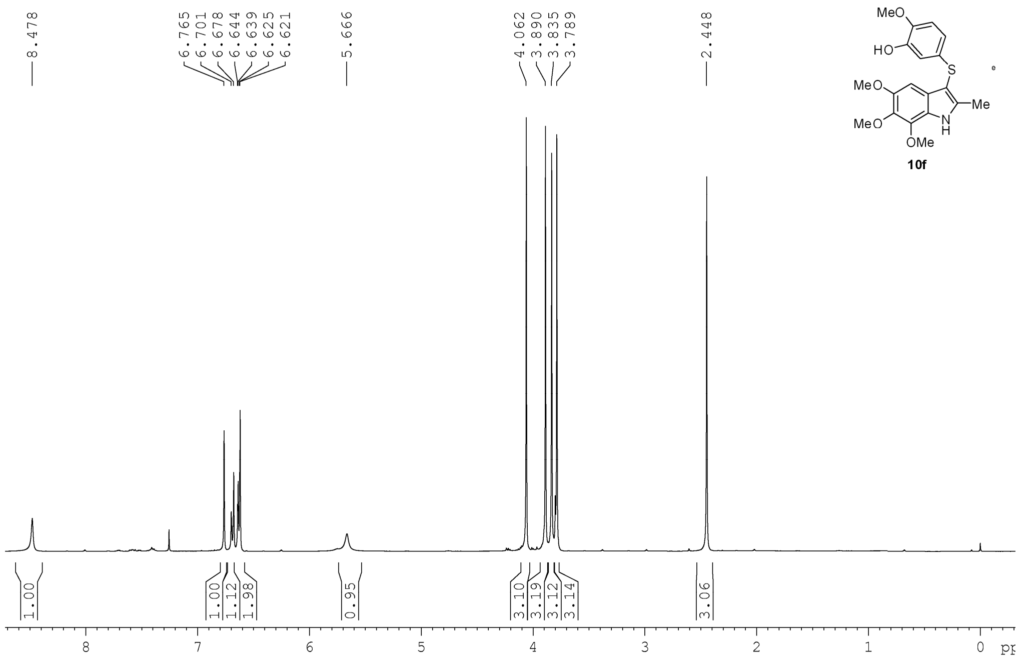

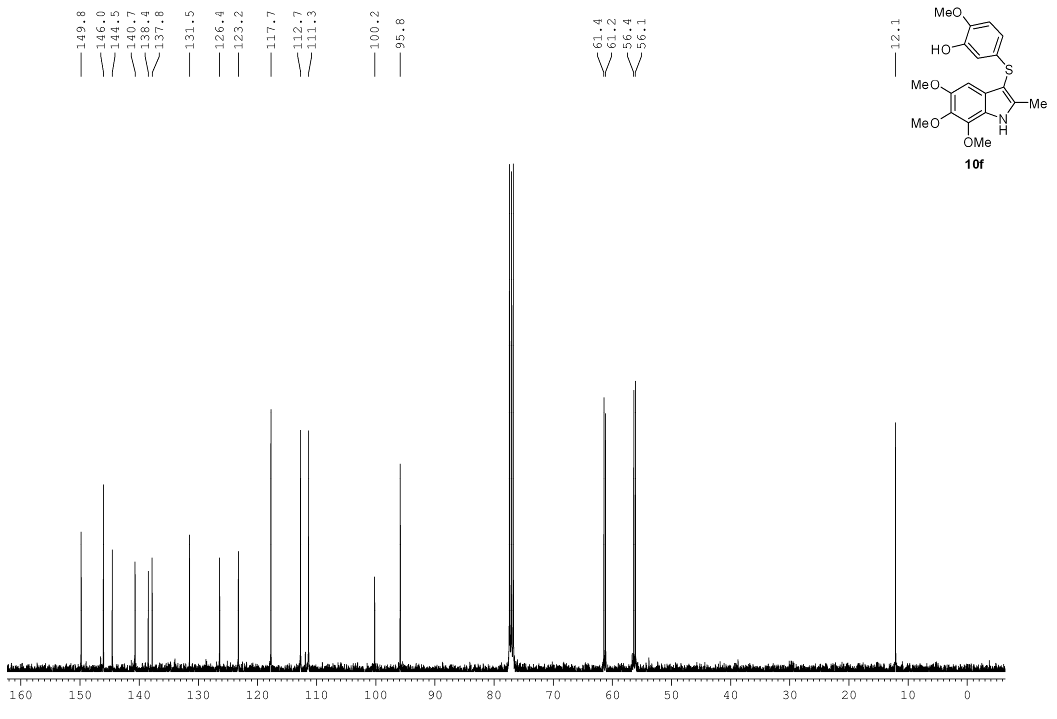

Ethyl 2-(3-(3-hydroxy-4-methoxyphenylthio)-5,6,7-trimethoxy-1*H*-indol-1-yl)acetate (**10g**)

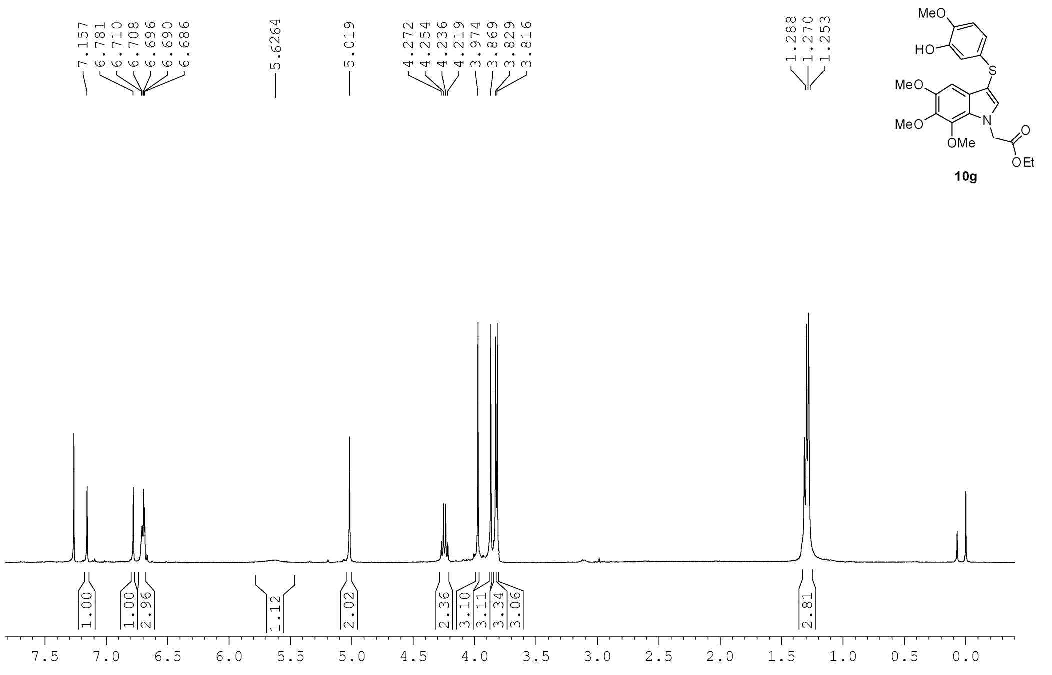

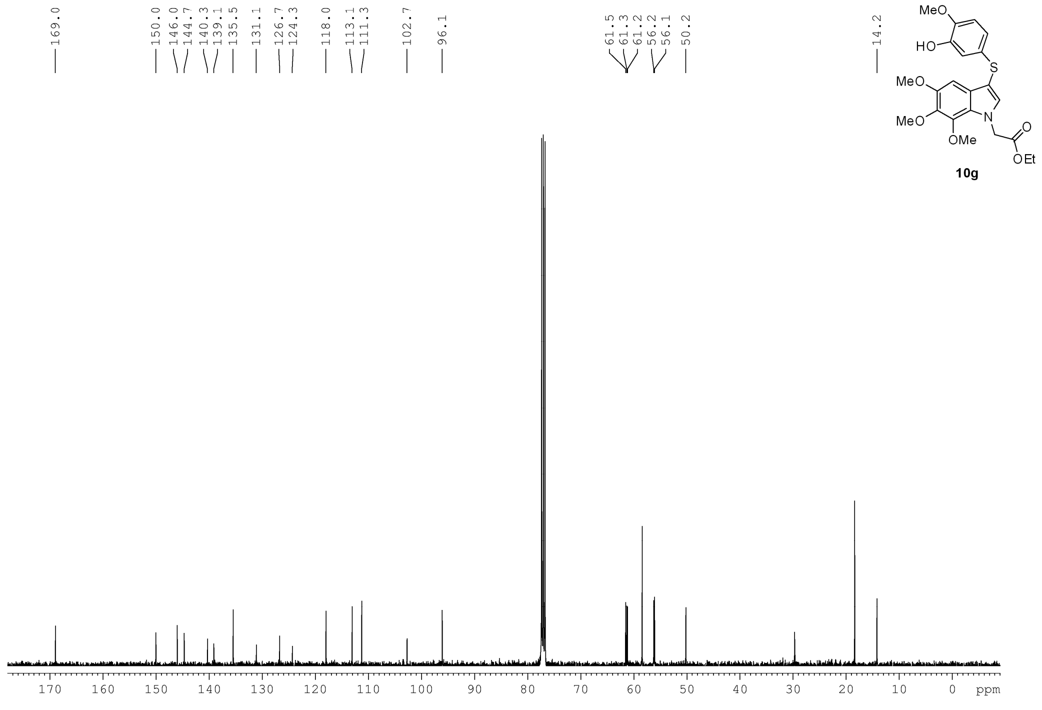

2-(3-(3-Hydroxy-4-methoxyphenylthio)-5,6,7-trimethoxy-1*H*-indol-1-yl)-1-morpholinoethanone **(10h)**

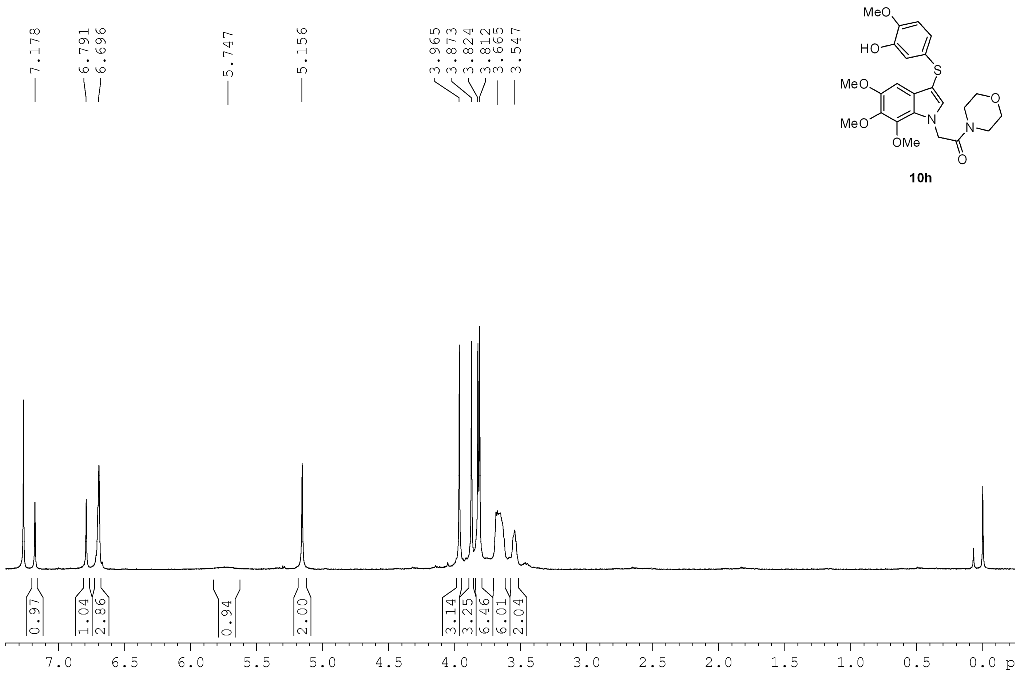

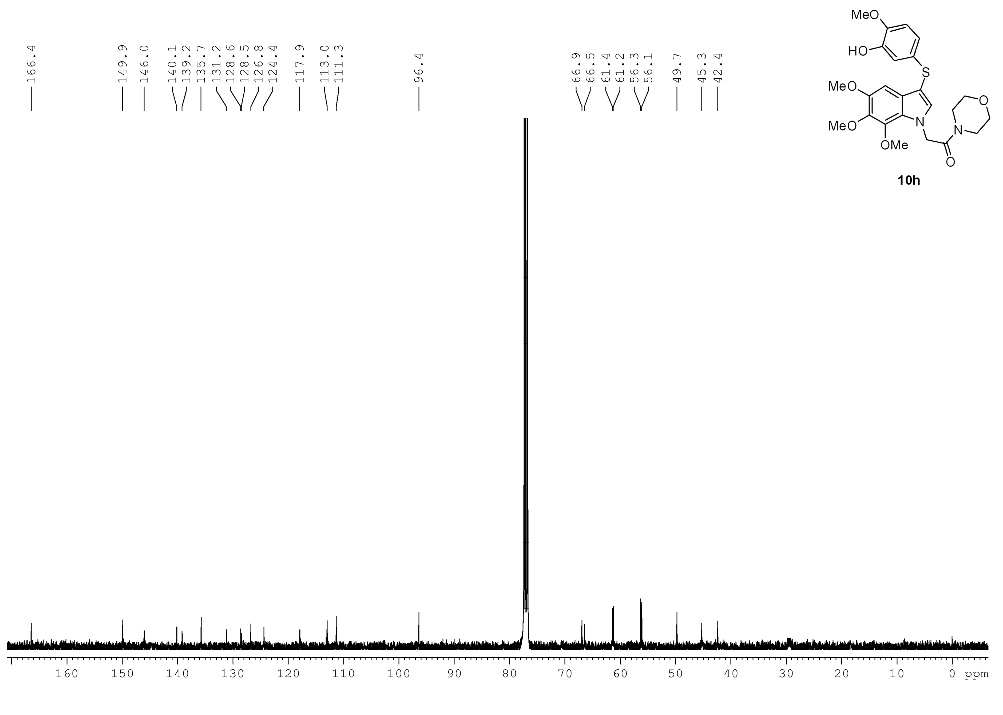

Supplement: Supplementary Information [file srep23986-s1.pdf]
